# Supplementary material for: Trends in the incidence and prevalence of dysphagia requiring medical attention among adults in South Korea, 2006–2016: A nationwide population study
Source: PLoS One. 2023 Jun 28;18(6):e0287512. doi: 10.1371/journal.pone.0287512 (PMC10306177; doi:10.1371/journal.pone.0287512)
Supplement: S1 Table — (DOCX) [file pone.0287512.s001.docx]

**S1 Table. Baseline characteristics according to dysphagia in the year of 2010.**

|  | **Dysphagia** | **Non-dysphagia** | **P-value** |
| --- | --- | --- | --- |
|  | (n = 39795) | (n = 39795) |  |
| **Age** |  |  |  |
| Average | 61.24±17.78 | | 1 |
| 20-29 | 2623 (6.6%) | |  |
| 30-39 | 3295 (8.3%) | |  |
| 40-49 | 4367 (10.9%) | |  |
| 50-59 | 6095 (15.3%) | |  |
| 60-69 | 7688 (19.3%) | |  |
| 70-79 | 9819 (24.7%) | |  |
| 80-89 | 5198 (13.1%) | |  |
| ≥90 | 710 (1.8%) | |  |
|  |  |  |  |
| **Sex (Male)** | 20629 (51.8%) | | 1 |
|  |  |  |  |
| **Income** |  |  |  |
| Low (25%) | 11349 (28.5%) | 10101 (25.4%) | <.001 |
|  |  |  |  |
| **Comorbidities** |  |  |  |
| Stroke | 19074 (47.9%) | 4530 (11.4%) | <.001 |
| ND | 11590 (29.1%) | 2224 (5.6%) | <.001 |
| Cancer | 4828 (12.1%) | 1545 (3.9%) | <.001 |
| COPD | 5179 (13.0%) | 1849 (4.7%) | <.001 |

**Notes**: Values are presented as number of people (% in each group). Dysphagia group was age- and sex- matched with non-dysphagia group.

ND: Neurodegenerative disease; COPD: Chronic obstructive pulmonary disease
