# Supplementary material for: Trends in the incidence and prevalence of dysphagia requiring medical attention among adults in South Korea, 2006–2016: A nationwide population study
Source: PLoS One. 2023 Jun 28;18(6):e0287512. doi: 10.1371/journal.pone.0287512 (PMC10306177; doi:10.1371/journal.pone.0287512)
Supplement: Attachment — Submitted filename: castejon S et al.pdf [file pone.0287512.s003.pdf]

Aging Clinical and Experimental Research
https://doi.org/10.1007/s40520-020-01707-9

SHORT COMMUNICATION

Association between anticholinergic burden and oropharyngeal dysphagia among hospitalized older adults

Santiago Castejón-Hernández[1,2,3] · Nadina Latorre-Vallbona[1,2] · Núria Molist-Brunet[1,2] · Dolors Cubí-Montanyà[1,2] · Joan Espaulella-Panicot[1,2]

Received: 12 June 2020 / Accepted: 1 September 2020
© Springer Nature Switzerland AG 2020

Abstract

Background Oropharyngeal dysphagia is an emerging age-related disorder that affects 23% of inpatients leading to malnutrition, dehydration, or aspiration pneumonia. Anticholinergic drugs can cause reduced peristalsis and dry mouth, both related to dysphagia.

Aim To determine the association between anticholinergic burden and oropharyngeal dysphagia in older inpatients.

Methods Retrospective descriptive observational study. There are 239 patients. Dysphagia diagnosis based on routine volume-viscosity swallow test. Characteristics: age, functional loss (instrumental and basic activities), frailty (Frail-VIG-Index), geriatric syndromes, polypharmacy, and anticholinergic-cognitive-burden scale at admission.

Results 25.5% of elderly patients diagnosed with dysphagia are more dependent and frailer than non-dysphagic patients. 83.6% scored ≥ 3 points on the ACB Scale [odds ratio: 4.46 (2.13–9.33)], which is statistically associated with dysphagia ($p < 0.001$).

Conclusion Patients with an ACB of ≥ 3 points at admission are more than four times as likely to develop oropharyngeal dysphagia. Evaluating anticholinergic burden routinely should be considered and, whenever possible, reduce it.

Keywords Anticholinergic burden · Dysphagia · Anticholinergic cognitive burden scale · Older inpatients

Introduction

Oropharyngeal dysphagia (OD) has been defined by the World Health Organization as the difficulty in forming or moving a bolus safely from the oral cavity to the esophagus. OD is an emerging disorder considered to be a new challenge for geriatricians as it is closely related to aging. It has been recognized as a geriatric syndrome [1] due to its high prevalence, association with aging, multiple risk factors, interactions with other geriatric syndromes, related complications, poor prognosis, and the need for a multidisciplinary and multifactorial approach.[2].

✉ Santiago Castejón-Hernández
santiagocastejon21@gmail.com

1 Acute Geriatric Unit (AGU), Geriatric Department, Hospital Universitari de Vic, Vic, Barcelona, Spain

2 Geriatric Department, Hospital Universitari de La Santa Creu, Vic, Barcelona, Spain

3 Santiago Castejón-Hernández, Rambla del Hospital, 52, 08500 Vic, Barcelona, Spain

OD prevalence varies depending on comorbidities, environment (hospital, intermediate care facility, home, etc.), and diagnostic methods: from 11.4% of patients living at home who self-manage to 23% of the patients in the acute geriatric unit (AGU) [3].

Changes in swallowing physiology related to aging (presbyphagia) and sarcopenia (sarcopenic dysphagia) make the development of OD complications more likely in older adults in the event of an acute emergency—such as hip fracture—or decompensated chronic illness causing them to be bedridden, lose functional capacity, etc. [3].

The decrease in the efficacy and safety of swallowing triggers OD complications, causing malnutrition and/or dehydration in the first case and aspiration and breathing complications, such as aspiration pneumonia, which is the most serious, in the second case [2, 3].

OD has also been described as a side-effect of some drugs, either by causing dystonia and dyskinesia (e.g., drugs with dopamine antagonist action such as metoclopramide) or by affecting the pharyngeal propulsive and clearance functions, as in the case of benzodiazepines [4].

The mechanism of action of anticholinergic drugs, typically prescribed for depression, behavioral disorders in dementia, Parkinson's disease (PD), epilepsy, gastrointestinal disorders, urinary incontinence, or to manage allergies [4–6], is blocking acetylcholine in both the central and peripheral nervous systems, and has different actions depending on the location of the receptor. Peripheral side-effects include constipation, urinary retention, bowel obstruction, peristaltic reduction, and dry mouth, among others [5–10].

Dry mouth (xerostomia), which is the most common peripheral side-effect of anticholinergic drugs and has been described as dose-dependent, results from the inhibition of salivation and may affect the ingestion and deglutition of food, leading to OD [4, 10, 11]. In older patients with multimorbidity and polypharmacy, the probability of exposure to anticholinergic drugs is higher, thus making this population more susceptible to their effects, which may be exacerbated by changes in pharmacodynamics and pharmacokinetics [6, 8]. Some studies have reported that 20–50% of older people take at least one medication with anticholinergic activity [7, 9].

Moreover, age, polypharmacy, comorbidities, or prior cognitive impairment have been described as risk factors for anticholinergic adverse events, which are sevenfold higher than in younger adults. For this reason, they have been identified as an inappropriate drug class for use by older people by the Beers Criteria [5, 6, 9].

Even so, there is no evidence to demonstrate the association between anticholinergic burden and dysphagia in older adults.

For that reason, our aim was to determine the association of anticholinergic burden to the development of oropharyngeal dysphagia in elderly people who are hospitalized.

Methods

In this retrospective, descriptive, observational study, we performed a systematic review of patients admitted to the Acute Geriatric Unit (AGU) between December 2016 and March 2017, collecting clinical information from their medical records in accordance with STROBE criteria.

The sample size was calculated based on 23% prevalence, 95% Confidence Interval (CI), and an estimated replacement rate of 10%, which resulted in 302 patients.

Patients who had previously presented with dysphagia (according to self-reported medical history concerning symptoms of dysphagia or who had been diagnosed prior to admission, including as a result of pharyngoesophageal stenosis) or with more than one hospitalization were excluded.

Baseline characteristics of all those included in the study were collected: age, gender, loss of both instrumental (IADL) and basic activities of daily living (BADL) (assessed using the Barthel Index), frailty score [based on the Frail-VIG Index (IF-VIG)—described by Amblàs et al. [12]], identification as complex chronic patient or as a patient with advanced chronic conditions (MACA) according to the NECPAL + tool [12] and presence of other geriatric syndromes (falls, bedsores, polypharmacy, etc.)

The admitting diagnosis of OD was based on the Volume-Viscosity Swallow Test (V-VST), which is routinely performed by the AGU unit on the first day of admission, considering that the diagnosis depends on the previous situation of the patient rather than on the admission itself. The test is done by the nursing staff, who are specifically trained to do it.

As per hospitalization-related variables, information about use of drugs potentially causing OD (such as sedatives, opioids or antipsychotics) was also collected. Anticholinergic burden during admission was measured using the Anticholinergic-cognitive-burden scale (ACBS) [13]. This variable was determined to be qualitative and scored from none/low (0–2 points on the ACBS) or high (3 or more points). The anticholinergic burden was calculated taking into account the chronical medication, that is, those drugs which the patient took at least 1 month before the day of admission according to the electronic prescription. The accumulative dose was not calculated, because there has not been clarified if the addition of different drugs with different scores has an anticholinergic summative effect or not.

The statistical analysis was performed with SPSS 24 software. The Chi-square test was used for an overall approach to compare the percentages among groups and to compare all percentage values among groups, while Student's *t* test and the Mann–Whitney *U* test were used in the comparative analysis (for both quantitative and qualitative-quantitative data with or without normal distribution, respectively). Statistical significance was set at $p < 0.05$.

Results

Of the 310 patients selected, 2 were excluded as they reported having previously had pharyngoesophageal stenosis, 6 for having been hospitalized more than once, and 63 patients due to a prior diagnosis or symptoms of dysphagia.

We finally included 239 patients (Fig. 1), 61 of whom were diagnosed with dysphagia after the V-VST test was performed, resulting in a prevalence of 25.5% in our cohort. The patients' ages ranged from 72 to 99 years (mean = 87.2 ± 4.4 SD) and 64.4% of them were female, with similar ratios between the two groups (dysphagic and not-dysphagic) and similar length of stay (between 6 and 7 days).

The 61 patients with OD were more dependent in both instrumental (more than 50% of these patients were

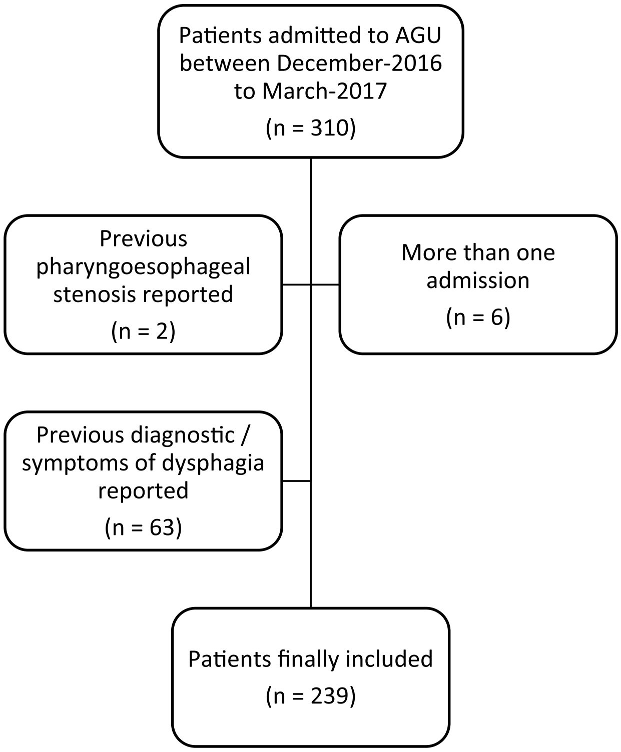

Fig. 1 Study flowchart of patient inclusion. *AGU* acute geriatric unit

unable to perform IADLs) and basic activities of daily living, scoring lower in the Barthel Index (BI) (average of 62.9 versus 77.1).

The median score of the dysphagic patients on the IF-VIG was 0.35 (upper limit of mild frailty) which is comparable with the non-OD cohort, which also ranked as mild but with lower score (0.28).

95.1% of the dysphagic patients had at least one other geriatric syndrome (apart from polypharmacy) and 70.5% of them were tagged as patients with advanced chronic conditions, showing a statistically significant association with dysphagia ($p < 0.001$). Furthermore, Barthel Index at discharge was significantly lower in patients who had OD than those without OD (25 and 55).

The rate of severe polypharmacy (≥ 10 medications) was 36.1% in patients diagnosed with OD, this percentage being higher than in non-OD patients. 49.3% of them scored high anticholinergic burden score ($p < 0.001$).

With regard to anticholinergic burden, it was higher in patients diagnosed with dysphagia: 83.6% had a score equal to or greater than 3 points versus 53.4% of the non-OD-patients, with an adjusted odds ratio (OR) (95% confidence interval) of 4.46 (2.13–9.33), showing statistical significance ($p < 0.001$) (Table 1).

Discussion

This study aimed to determine whether there is an association between anticholinergic burden and dysphagia.

Anticholinergic drugs have been linked to other geriatric syndromes such as delirium, bedsores, falls, and even cognitive impairment [3, 5] and may affect all stages of the swallowing process by causing aspiration, either as a result of their sedative effect or by affecting mouth dryness [4, 10, 11]. We included anticholinergic burden in our study, because it seems to be a relevant issue to take into consideration, as it is a potentially modifiable factor often overlooked in frail patients. In our study, we found mild frailty according to the VIG-Frail Index, with little difference between dysphagic and non-dysphagic patients, although frailty was higher in those diagnosed with OD.

With respect to anticholinergic burden, there is no consensus as to which scale is the best, but most of them assign a score of 0–3 to different drugs. In the case of the ACBS, drugs are classified as having a low (1 point), moderate (2 points), or high (3 points) anticholinergic burden depending on their anticholinergic action, which is mostly measured by in vitro or serum anticholinergic activity [8, 10, 13]. If they have anticholinergic activity or in vitro affinity to muscarinic receptors but no clinical cognitive effects, they are assigned a score of 1 (low activity). Those with established and clinically relevant anticholinergic effects based on blood–brain penetration have been assigned a score of 2 points (definitely anticholinergic, moderate activity). And, if they fulfill these characteristics and have also been described as a cause of delirium, they score 3 (definitely anticholinergic, high activity) [5, 13].

80% of drugs identified with low anticholinergic activity are used very frequently, including furosemide, citalopram, metformin, warfarin, and digoxin, among others. Drugs with moderate-to-high burden include amitriptyline, quetiapine, haloperidol, and paroxetine [9, 13].

Kersten et al. have mentioned on their paper that the dose–response relationship with an ADS score ≥ 3 has not previously been investigated, but this higher ADS score could be a significant predictor of cognitive impairment, although they were not able to demonstrate it. Thus, they propose a non-additive effect of the combination of multiple anticholinergic drugs maybe because of the pharmacodynamics brain effects of these drugs, making more difficult to establish a linear pattern between ADS score and side-effects of anticholinergic drugs [14].

It has been reported that 23–35% of older adults take at least one drug with an anticholinergic burden score of 3 points, which means an increased risk for both peripheral and central anticholinergic effects as defined by Lieberman et al. [15].

Table 1 Characteristics of study population

Characteristics	Total ($n=239$)	Dysphagia ($n=61$)	No dysphagia ($n=178$)	OR (95% CI)	p value
Women	154 (64.4%)	38 (62.3%)	116 (65.2%)	NS	0.686
Age (years), mean ± SD	87.2 ± 4.4	87.7 ± 4.4	87.0 ± 4.4	NA	0.309[a]
LOS (days), mean ± SD	6.35 ± 4.4	6.95 ± 3.2	6.15 ± 4.7	NA	0.218[a]
Loss of IADLs					
All	98 (41%)	33 (54.1%)	65 (36.5%)	NA	0.003*
Some	46 (19.2%)	15 (24.6%)	31 (17.4%)		
None	95 (39.7%)	13 (21.3%)	82 (46.1%)		
Previous BI, mean ± SD	73.5 ± 25.5	62.9 ± 27.5	77.1 ± 23.7	NA	< 0.001[b*]
Cognitive level					
No impairment	145 (60.7%)	31 (50.8%)	114 (64%)	NA	0.090
Impairment suspected	26 (10.9%)	5 (8.2%)	21 (11.8%)		
Mild cognitive impairment	31 (13%)	12 (19.7%)	19 (10.7%)		
Dementia	37 (15.5%)	13 (21.3%)	24 (13.5%)		
Polypharmacy				NA	0.312
< 5 drugs	39 (16.3%)	7 (11.5%)	32 (18.0%)		
5–9 drugs	129 (54%)	32 (52.5%)	97 (54.5%)		
≥ 10 drugs	71 (29.7%)	22 (36.1%)	49 (27.5%)		
Anticholinergic burden at admission					
None/Low	93 (38.9%)	10 (16.4%)	83 (46.6%)	4.46 {2.13–9.33}	< 0.001*
High	146 (61.1%)	51 (83.6%)	95 (53.4%)		
Advanced chronic conditions	115 (48.1%)	43 (70.5%)	72 (40.4%)	3.52 {1.88–6.58}	< 0.001*
Frail-VIG index, mean ± SD	0.30 ± 0.15	0.35 ± 0.14	0.28 ± 0.15	NA	0. 002[a*]
Geriatric syndromes	213 (89.1%)	58 (95.1%)	155 (87.1%)	2.87 {0.83–9.92}	0.083
BI at discharge, mean ± SD	47.1 ± 31.4	24.8 ± 24.7	54.7 ± 29.9	NA	< 0.001[b*]

BI Barthel index, *CI* confidence interval, *IADLs* instrumental activities of daily living, *LOS* length of stay, *NA* not applicable, *NS* not significant, *OR* odds ratio, *SD* standard deviation

*statistically significant

[a]Calculated using t-student, [b]Calculated using U Mann–Whitney

72.8% of elderly persons are exposed to strong anticholinergic drugs [5, 6] and those with higher intake of these drugs have greater risk of side-effects with lower benefits from treatment, apart from more potential for drug–drug interactions [6]. In our case, 61.1% of our patients had an ACBS score of three points, with this percentage being higher in our dysphagic patients, reaching 83.6%.

Some studies have reported that a systematic review of anticholinergic burden in clinical practice—especially in frail patients—could lead to a decrease in the use of these drugs, starting by decreasing to as low a dose as possible if it is imperative to treatment. Other options are to eliminate unnecessary medications (to try to avoid drug interactions) or switch to another drug with lower or no anticholinergic burden [7, 15].

Although xerostomia and dysphagia are listed as possible side-effects of anticholinergic drugs, the association of anticholinergic burden and OD has not been established. Kersten et al. observed that ADS score was significantly associated with peripheral anticholinergic side-effects: higher serum anticholinergic activity (1.2-fold) and 0.7-fold lower saliva production in subjects with ADS scores ≥ 6 compared with those with an ADS score of 3 [14].

However, in our study, we found an OR of 4.46, which shows a possible relationship between exposition to anticholinergic burden and the development of dysphagia during hospitalization.

Hence, further studies including interventions to decrease the anticholinergic burden are needed to demonstrate its involvement in the development of OD.

As limitations of our study, we should note the collection of retrospective data. This may introduce a selection bias as a result of the study population not being representative of the target population, since hospitalized patients may be more frail. The absence of any intervention (no deprescribing or later assessment of the ACB after discharge) could be another weakness. Even admission due to decompensation of a chronic condition or for a new disease could be involved in the development of dysphagia, although it is difficult to establish the cause–effect relationship between

different risk factors and dysphagia. Another issue is what happens with people who have ACB scores of 1 or 2 points and if the deprescribing of anticholinergic drugs in both groups is involved in the lower development of dysphagia. Finally, we should mention that any of the scales have been identified as ideal to measure the anticholinergic burden in all circumstances, but we justified the choice of the ACBS, because it has been described as suited for implementation in the observational studies where the anticholinergic exposure must be quantified. Also, this scale includes the largest number of medications and it was validated with adverse clinical outcomes as Lozano-Ortega et al. have described in their review [16]. As strengths, our study included the ACBS as a tool for measuring anticholinergic burden, and we found an association between this scale and the onset of dysphagia in older adults, although this association may not be directly clear, because dysphagic patients are more frail, and have multiple chronic conditions and less functionality.

Conclusions

Oropharyngeal dysphagia is a frequent geriatric syndrome in the AGU affecting one out of four inpatients (25.5%) with multifactorial causes, including potentially modifiable factors such as drugs.

An anticholinergic burden of more than three points has been associated more than four times as much with the development of oropharyngeal dysphagia during hospitalization, although we cannot confirm if there is a direct association between both factors due to the multifactorial origin of dysphagia in elderly persons.

Therefore, clinicians should be aware of the side-effects of anticholinergic drugs and familiarize themselves with anticholinergic risk assessment tools. For this reason, anticholinergic burden should be evaluated routinely when anticholinergic drugs are prescribed (including those that have a score of one point) and reducing this burden whenever possible should be considered.

Compliance with ethical standards

Conflict of interest The authors declare that they have no conflict of interest.

Ethical standards The authors certify that they comply with the ethical guidelines for authorship and publishing of the Aging Clinical and Experimental Research Journal.

Ethical approval and Informed consent As our paper has been done as a retrospective collecting data, based on clinical research on a database, we didn't collect any informed consent. Also, as by the retrospective characteristics of this study, the study was commented to our Ethical committee, who decided it was not essential the approval of this Committee.

References

1. Martín A, Ortega O, Clavé P (2018) Disfagia orofaríngea, un nuevo síndrome geriátrico. Rev Esp Geriatr Gerontol 53:3–5
2. Almirall J, Rofes L, Serra-Prat M et al (2013) Oropharyngeal dysphagia is a risk factor for community-acquired pneumonia in the elderly. Eur Respir J 41:923–928
3. Cabre M, Serra-Prat M, Force L et al (2014) Oropharyngeal dysphagia is a risk factor for readmission for pneumonia in the very elderly persons: observational prospective study. J Gerontol Ser A Biol Sci Med Sci 69A:330–337
4. Schindler JS, Kelly JH (2002) Swallowing disorders in the elderly. Laryngoscope 112:589–602
5. Richardson K, Fox C, Maidment I et al (2018) Anticholinergic drugs and risk of dementia: case-control study. BMJ 361:k1315
6. Joung K, Shin J, Cho S (2019) Features of anticholinergic prescriptions and predictors of high use in the elderly: population-based study. Pharmacoepidemiol Drug Saf 28:1591–1600
7. Fox C, Smith T, Maidment I et al (2014) Effect of medications with anti-cholinergic properties on cognitive function, delirium, physical function and mortality: a systematic review. Age Ageing 43:604–615
8. Pierce H, Thomas D, Asfaw T et al (2019) Anticholinergic burden in the elderly population: an emerging concern. Eur Urol 76:7–8
9. Salahudeen MS, Nishtala PS, Duffull SB (2016) The influence of patient characteristics on anticholinergic events in older people. Dement Geriatr Cogn Dis Extra 5:530–541
10. Nishtala PS, Salahudeen MS, Hilmer SN (2016) Anticholinergics: theoretical and clinical overview. Expert Opin Drug Saf 15:753–768
11. Cook IJ (2009) Oropharyngeal dysphagia. Gastroenterol Clin North Am 38:411–431
12. Amblàs-Novellas J, Martori JC, Molist Brunet N et al (2017) Índice frágil-VIG: diseño y evaluación de un índice de fragilidad basado en la Valoración integral geriátrica. Rev Esp Geriatr Gerontol 52:119–127
13. Boustani M, Campbell N, Munger S et al (2008) Impact of anticholinergics on the aging brain: a review and practical application. Aging Health 4:311–320
14. Kersten H, Molden E, Willumsen T et al (2013) Higher anticholinergic drug scale (ADS) scores are associated with peripheral but not cognitive markers of cholinergic blockade. Cross sectional data from 21 Norwegian nursing homes. Br J Clin Pharmacol 75(3):842–849
15. Lieberman JA (2004) Managing anticholinergic side effects. Prim Care Companion J Clin Psychiatry 6:20–23
16. Lozano-Ortega G, Johnston KM, Cheung A et al (2020) A review of published anticholinergic scales and measures and their applicability in database analyses. Arch Gerontol Geriatr 87:103885

Publisher's Note Springer Nature remains neutral with regard to jurisdictional claims in published maps and institutional affiliations.
