# Supplementary material for: Trends in the incidence and prevalence of dysphagia requiring medical attention among adults in South Korea, 2006–2016: A nationwide population study
Source: PLoS One. 2023 Jun 28;18(6):e0287512. doi: 10.1371/journal.pone.0287512 (PMC10306177; doi:10.1371/journal.pone.0287512)
Supplement: Attachment — Submitted filename: PONE-D-22-35490R1 - SCH.pdf [file pone.0287512.s005.pdf]

PLOS ONE

Trends in the incidence and prevalence of dysphagia requiring medical attention among adults in South Korea, 2006–2016: A nationwide population study

--Manuscript Draft--

**Manuscript Number:**	PONE-D-22-35490R1
**Article Type:**	Research Article
**Full Title:**	Trends in the incidence and prevalence of dysphagia requiring medical attention among adults in South Korea, 2006–2016: A nationwide population study
**Short Title:**	Trends of dysphagia in South Korea
**Corresponding Author:**	Won-Seok Kim<br>Seoul National University Bundang Hospital<br>Seongnam-si, Gyeonggi-do KOREA, REPUBLIC OF
**Keywords:**	Dysphagia; Incidence; prevalence; Mortality; Hazard Ratio
**Abstract:**	The prevalence of dysphagia is increasing, resulting in socioeconomic burden, but previous reports have only been based on a specific population. Therefore, we aimed to investigate the nationwide incidence and prevalence of dysphagia requiring medical attention to provide adequate information for healthcare planning and resource allocation. In this nationwide retrospective cohort study, the data of adults aged ≥20 years recorded from 2006 to 2016 were sourced from the Korean National Health Insurance Service database. Medical claim codes based on ICD-10-CM were used to define dysphagia and possible causes. The annual incidence and prevalence of dysphagia were calculated. Cox regression was used to estimate dysphagia risk in people with possible dysphagia etiology. Survival analysis was performed to estimate the mortality and hazard ratio of dysphagia. The crude annual incidence of dysphagia increased continuously from 7.14 in 2006 to 15.64 in 2016. The crude annual prevalence of dysphagia in 2006 was 0.09% and increased annually to 0.25% in 2016. Stroke (odds ratio [OR]: 7.86, 95% confidence interval [CI]: 5.76–6.68), neurodegenerative disease (OR: 6.20, 95% CI: 5.76–6.68), cancer (OR: 5.59, 95% CI: 5.17–6.06), and chronic obstructive pulmonary disease (OR: 2.94, 95% CI: 2.71–3.18) were associated with a high risk of dysphagia. The mortality in the dysphagia group was 3.12 times higher than that in the non-dysphagia group (hazard ratio: 3.12, 95% CI: 3.03–3.23). The incidence and prevalence of dysphagia requiring medical attention are increasing annually. The increasing trend was conspicuous in the geriatric group. The presence of stroke, neurodegenerative disease, cancer, and chronic obstructive pulmonary disease is associated with a high risk of dysphagia. Therefore, adequate screening, diagnosis, and management of dysphagia in the older population must be emphasized in geriatric healthcare.
**Order of Authors:**	SuYeon Kwon
	Seungwoo Cha
	Junsik Kim
	Kyungdo Han
	Nam-Jong Paik
	Won-Seok Kim
**Response to Reviewers:**	We greatly appreciate the efforts of the reviewers for their constructive comments in evaluating our manuscript. Each comment has been carefully considered and responses have been provided for each point.
**Additional Information:**	
**Question**	**Response**
**Financial Disclosure**<br><br>Enter a financial disclosure statement that	This work was supported by Seoul National University Bundang Hospital (https://www.snubh.org) Research Fund (No. 06-2018-140). The funders did not play a role in the study design, data collection and analysis, decision to publish, or

describes the sources of funding for the work included in this submission. Review the submission guidelines for detailed requirements. View published research articles from *PLOS ONE* for specific examples.<br><br>This statement is required for submission and **will appear in the published article** if the submission is accepted. Please make sure it is accurate.<br><br>**Unfunded studies**<br>Enter: *The author(s) received no specific funding for this work.*<br><br>**Funded studies**<br>Enter a statement with the following details:<br>• Initials of the authors who received each award<br>• Grant numbers awarded to each author<br>• The full name of each funder<br>• URL of each funder website<br>• Did the sponsors or funders play any role in the study design, data collection and analysis, decision to publish, or preparation of the manuscript?<br>• **NO** - Include this sentence at the end of your statement: *The funders had no role in study design, data collection and analysis, decision to publish, or preparation of the manuscript.*<br>• **YES** - Specify the role(s) played.<br><br>* typeset	preparation of the manuscript.
**Competing Interests**<br><br>Use the instructions below to enter a competing interest statement for this submission. On behalf of all authors, disclose any competing interests that could be perceived to bias this work—acknowledging all financial support and any other relevant financial or non-financial competing interests.<br><br>This statement is required for submission and **will appear in the published article** if the submission is accepted. Please make sure it is accurate and that any funding	The authors have declared that no competing interests exist.

Powered by Editorial Manager® and ProduXion Manager® from Aries Systems Corporation

sources listed in your Funding Information later in the submission form are also declared in your Financial Disclosure statement.<br><br>View published research articles from *PLOS ONE* for specific examples.<br><br>**NO authors have competing interests**<br><br>Enter: *The authors have declared that no competing interests exist.*<br><br>**Authors with competing interests**<br><br>Enter competing interest details beginning with this statement:<br><br>*I have read the journal's policy and the authors of this manuscript have the following competing interests: [insert competing interests here]*<br><br>* typeset	
**Ethics Statement**<br><br>Enter an ethics statement for this submission. This statement is required if the study involved:<br><br>• Human participants<br>• Human specimens or tissue<br>• Vertebrate animals or cephalopods<br>• Vertebrate embryos or tissues<br>• Field research<br><br>Write "N/A" if the submission does not require an ethics statement.<br><br>General guidance is provided below. Consult the submission guidelines for detailed instructions. **Make sure that all information entered here is included in the Methods section of the manuscript.**	This study was exempt from review by the Seoul National University Bundang Hospital Institutional Review Board (X-1807/483-904), complying with the requirements of the Declaration of Helsinki. Data from the Korean NHIS were fully anonymized for analyses, and the need for obtaining informed consent was waived.

Powered by Editorial Manager® and ProduXion Manager® from Aries Systems Corporation

**Format for specific study types**<br><br>**Human Subject Research (involving human participants and/or tissue)**<br>• Give the name of the institutional review board or ethics committee that approved the study<br>• Include the approval number and/or a statement indicating approval of this research<br>• Indicate the form of consent obtained (written/oral) or the reason that consent was not obtained (e.g. the data were analyzed anonymously)<br><br>**Animal Research (involving vertebrate animals, embryos or tissues)**<br>• Provide the name of the Institutional Animal Care and Use Committee (IACUC) or other relevant ethics board that reviewed the study protocol, and indicate whether they approved this research or granted a formal waiver of ethical approval<br>• Include an approval number if one was obtained<br>• If the study involved *non-human primates*, add *additional details* about animal welfare and steps taken to ameliorate suffering<br>• If anesthesia, euthanasia, or any kind of animal sacrifice is part of the study, include briefly which substances and/or methods were applied<br><br>**Field Research**<br><br>Include the following details if this study involves the collection of plant, animal, or other materials from a natural setting:<br>• Field permit number<br>• Name of the institution or relevant body that granted permission	
**Data Availability**<br><br>Authors are required to make all data underlying the findings described fully available, without restriction, and from the time of publication. PLOS allows rare exceptions to address legal and ethical concerns. See the PLOS Data Policy and FAQ for detailed information.	No - some restrictions will apply

A Data Availability Statement describing where the data can be found is required at submission. Your answers to this question constitute the Data Availability Statement and **will be published in the article**, if accepted.<br><br>**Important:** Stating 'data available on request from the author' is not sufficient. If your data are only available upon request, select 'No' for the first question and explain your exceptional situation in the text box.<br><br>Do the authors confirm that all data underlying the findings described in their manuscript are fully available without restriction?	
**Describe where the data may be found in full sentences. If you are copying our sample text, replace any instances of XXX with the appropriate details.**<br><br>• If the data are **held or will be held in a public repository**, include URLs, accession numbers or DOIs. If this information will only be available after acceptance, indicate this by ticking the box below. For example: *All XXX files are available from the XXX database (accession number(s) XXX, XXX.).*<br>• If the data are all contained **within the manuscript and/or Supporting Information files**, enter the following: *All relevant data are within the manuscript and its Supporting Information files.*<br>• If neither of these applies but you are able to provide **details of access elsewhere**, with or without limitations, please do so. For example:<br><br>*Data cannot be shared publicly because of [XXX]. Data are available from the XXX Institutional Data Access / Ethics Committee (contact via XXX) for researchers who meet the criteria for access to confidential data.*<br><br>*The data underlying the results presented in the study are available from (include the name of the third party*	Data cannot be shared publicly because they belong to the National Health Insurance Service (NHIS). There are ethical restrictions on sharing a data set because data contain potentially identifying or sensitive patient information.To request data from NHIS(access number NHIS-2018-1-331), researchers have to apply during the recruitment period and submit a research proposal. The committee reviews the proposals and then selects a few researchers to use and analyze the data. Data access applications for the national health insurance data are available on the NHIS data sharing website (https://nhiss.nhis.or.kr/bd/ab/bdaba000eng.do). Raw data was available to the researchers upon reasonable academic request and with the permission of the Korean NHIS Institutional Data Access (http://nhiss.nhis.or.kr). The authors had no special access privileges.

and contact information or URL).<br>• This text is appropriate if the data are owned by a third party and authors do not have permission to share the data.<br><br>* typeset	
Additional data availability information:	

Powered by Editorial Manager® and ProduXion Manager® from Aries Systems Corporation

Manuscript

Click here to access/download;Manuscript;Manuscript_Dysphagia_PONE

Trends in the incidence and prevalence of dysphagia requiring medical attention among adults in South Korea, 2006–2016: A nationwide population study

Short title: Trends of dysphagia in South Korea

SuYeon Kwon[1], Seungwoo Cha[2], Junsik Kim[2], Kyungdo Han[3], Nam-Jong Paik[2], Won-Seok Kim[2*]

[1]Department of Rehabilitation Medicine, Ewha Woman's University Seoul Hospital, School of Medicine, Seoul, Republic of Korea

[2]Department of Rehabilitation Medicine, Seoul National University College of Medicine, Seoul National University Bundang Hospital, Gyeonggi-do, Republic of Korea

[3]Department of Statistics and Actuarial Science, Soongsil University, Seoul, Republic of Korea

***Corresponding author**

E-mail: wondol77@gmail.com (WSK)

Keywords: Dysphagia, Incidence, Prevalence, Mortality, Hazard Ratio

Abstract

The prevalence of dysphagia is increasing, resulting in socioeconomic burden, but previous reports have only been based on a specific population. Therefore, we aimed to investigate the nationwide incidence and prevalence of dysphagia requiring medical attention to provide adequate information for healthcare planning and resource allocation. In this nationwide retrospective cohort study, the data of adults aged ≥20 years recorded from 2006 to 2016 were sourced from the Korean National Health Insurance Service database. Medical claim codes based on ICD-10-CM were used to define dysphagia and possible causes. The annual incidence and prevalence of dysphagia were calculated. Cox regression was used to estimate dysphagia risk in people with possible dysphagia etiology. Survival analysis was performed to estimate the mortality and hazard ratio of dysphagia. The crude annual incidence of dysphagia increased continuously from 7.14 in 2006 to 15.64 in 2016. The crude annual prevalence of dysphagia in 2006 was 0.09% and increased annually to 0.25% in 2016. Stroke (odds ratio [OR]: 7.86, 95% confidence interval [CI]: 5.76–6.68), neurodegenerative disease (OR: 6.20, 95% CI: 5.76–6.68), cancer (OR: 5.59, 95% CI: 5.17–6.06), and chronic obstructive pulmonary disease (OR: 2.94, 95% CI: 2.71–3.18) were associated with a high risk of dysphagia. The mortality in the dysphagia group was 3.12 times higher than that in the non-dysphagia group (hazard ratio: 3.12, 95% CI: 3.03–3.23). The incidence and prevalence of dysphagia requiring medical attention are increasing annually. The increasing trend was conspicuous in the geriatric group. The presence of stroke, neurodegenerative disease, cancer, and chronic obstructive pulmonary disease is associated with a high risk of dysphagia. Therefore, adequate

screening, diagnosis, and management of dysphagia in the older population must be emphasized in geriatric healthcare.

Introduction

Swallowing is the process of passing solid and liquid food from the oral cavity, pharynx, and esophagus into the stomach with appropriate coordination [1]. Normal physiological swallowing is important not only for adequate nutrition and hydration but also for prevention of complications, such as aspiration pneumonia and asphyxia [2,3]. The World Health Organization has defined dysphagia as the difficulty in forming or moving a bolus safely from the oral cavity to the esophagus [4] and has recognized it as a medical disability associated with increased morbidity, mortality, and cost of treatment [1,3].

The swallowing process can be disrupted by diverse diseases such as stroke, traumatic brain injury, neurodegenerative disease (ND), motor neuron diseases, and cancers [5,6]. Since these diseases are increasing with the aging of the population, the incidence of dysphagia, as one of the associated complications, is expected to increase accordingly [7,8]. In addition, aging is associated with dysphagia due to muscle wasting, delayed swallowing reflex, and neuromuscular incoordination [2,3,9,10]. Dysphagia is becoming more common in the geriatric population [8] as life expectancy increases. The prevalence of dysphagia in the geriatric population has been reported variably in different countries. In the United States, the prevalence of dysphagia in adults over 50 years of age ranges from 11.4% to 22% [5,11], and that in the geriatric population living in the community is 33% [12]. In Europe, the prevalence of dysphagia in the geriatric population living in the community ranges from 30% to 40%, and that in hospital/ institutional settings ranges from 25.5% to 60% [4,7]. In Asia, the prevalence of dysphagia in the geriatric population living in the community ranges from 13.8% to

33.7% [13,14]. A meta-analysis and systematic review of the multinational studies reported that dysphagia affects 30% of community-dwelling geriatric population, almost 50% of geriatric patients, and above 50% of nursing home residents [15]. Therefore, diagnosing and managing dysphagia is an important issue in geriatric healthcare.

The previously reported prevalence of dysphagia is not representative of the entire population because most of these reports have come from cross-sectional studies with small sample sizes, specific diseases (e.g., stroke, ND, cancer) [16–20], and specific healthcare settings (hospitalized/institution settings) with specific age groups (healthy or frail geriatric population) [12–14]. Hence, there is a lack of nationwide data on the prevalence, incidence, and mortality of dysphagia to establish relevant healthcare policies on the evaluation and management of dysphagia.

Therefore, the primary purpose of this study was to investigate the nationwide trend of the incidence and prevalence of dysphagia requiring medical attention from a large, representative, population-based cohort, using claim data from the Korean National Health Insurance Service (NHIS) database from 2006 to 2016. We further investigated the possible diseases associated with dysphagia, and the risk of long-term mortality in dysphagic individuals compared to non-dysphagic individuals.

Materials and Methods

Data source and study population

The data source for this study is the NHIS database of South Korea. The NHIS has granted access to researchers since 2014. The NHIS is a universal social health insurance that provides healthcare coverage and medical aid to most of the Korean population (97.2% and 2.8%, respectively) [21]. Therefore, the NHIS presumptively has all records of medical service claims and sociodemographic information of the entire Korean population. The claim codes used in the Korean NHIS are based on the International Classification of Disease-Tenth Revision, Clinical Modification (ICD-10-CM). The complete data of the adult population aged ≥20 years (inpatient and outpatient medical service claims and sociodemographic information) from 2006 to 2016 were extracted. This study was exempt from review by the Seoul National University Bundang Hospital Institutional Review Board (X-1807/483-904), complying with the requirements of the Declaration of Helsinki. Data from the Korean NHIS were fully anonymized for analyses, and the need for obtaining informed consent was waived.

Definition of dysphagia and diseases that may have caused dysphagia

To define dysphagia, diagnostic codes and medical claim codes related to diagnosis or treatment of dysphagia were used. If a person matched at least one of the following criteria, the individual was assumed to have dysphagia: 1) presence of the diagnostic code for dysphagia (R13), 2) swallowing therapies (MX141) at least twice within 1 month, 3) instrumental swallowing tests (E7011, E7012) such as videofluoroscopic swallowing studies (VFSS) or fiberoptic endoscopic evaluation of swallowing (FEES) at least twice within 3 months, 4) nasogastric tube insertions

(Q2621, Q2622) at least twice within 3 months, or 5) procedure code claims for percutaneous gastrostomy (M6730, Q2612) at least once. In summary, the operational definition tried to include dysphagic people who received medical attention for dysphagia diagnosis or management during admission or outpatient visits.

Medical claim codes based on ICD-10-CM were used to define the diseases that may have caused dysphagia: 1) stroke as diagnostic codes of I63 and I64 and brain imaging (computed tomography or magnetic resonance imaging) during admission, 2) ND as diagnostic codes claimed for Parkinson’s disease (G20 and V124) or those claimed for dementia (F00-03, G30, G31, G231, G310, G318, and F107), 3) cancer as diagnostic code of C codes (C00-14, C15-25, C32-34, C50, C53, C54, C56, C61, C62, C64, C67, C70-72, C73, C81-86, C90-95) and V193, and 4) chronic obstructive pulmonary disease (COPD) as diagnostic codes of J41-J44. V193 and V194 are specific deductible insurance codes for cancer and Parkinson's disease respectively in Korea. These four diseases were selected because they have been associated with dysphagia in previous studies [18,19,22].

Other variables

Other epidemiologic factors including age, sex, and income, were obtained and stratified into quartiles for statistical analyses. Data regarding death from various causes were obtained from the NHIS database linked to Statistics Korea, which records deaths of Korean citizens.

Statistics

The crude annual incidence was calculated by dividing the number of new patients with dysphagia each year by 10,000 person-years at risk. People diagnosed with dysphagia before 2006 were excluded as they would not represent new cases. The age-adjusted incidence and the incidence of dysphagia in each age group were calculated for each year to control for different age distributions throughout the years. The crude annual prevalence was calculated by dividing the number of people with dysphagia each year by the total of 10,000 people. Individuals who did not meet the operational definition of dysphagia in that year were excluded, even though they might have met the definition in the previous years. The age-adjusted prevalence and prevalence of dysphagia in each age group were also calculated.

To investigate the risk of dysphagia in possible common etiologies of dysphagia and the risk of mortality in dysphagia, data of people who were defined as having dysphagia for the first time in 2010 (n = 39795) were extracted and these individuals were followed up until 2016; these data were compared with the data of the age- and sex-matched population without dysphagia (n = 39795). The chi-square test was used to compare categorical variables between the dysphagia and non-dysphagia groups. Logistic regression analysis was used to determine the risk of dysphagia in each possible common etiology of dysphagia (stroke, ND, cancer, and COPD) in 2010. Adjustments for age, sex, low-income status, and comorbidities were made. Multivariate adjusted Cox proportional hazard regression analysis was used to identify the risk of mortality in the dysphagia group. Time-to-event analysis was used to estimate the mortality rate, and a Kaplan–Meier survival curve was plotted and

compared using the log-rank test. All statistical analyses were performed using SAS version 9.4 (SAS Institute Inc., Cary, NC, USA). Statistical significance was set at $P < 0.05$.

Results

Incidence of dysphagia

The crude annual incidence of dysphagia increased every year from 7.14 in 2006 to 15.64 in 2016, and the age-adjusted annual incidence showed a similar increasing trend (**Fig. 1A-C**). In addition, the annual incidence of dysphagia increased more prominently in the geriatric group aged >70 years. (**Fig. 1D-F**).

Fig. 1. **Incidence of dysphagia between 2006 and 2016 (A) total, (B) male, (C) female, (D) total according to age group, (E) male according to age group, (F) female according to age group**.

Prevalence of dysphagia

The crude annual prevalence of dysphagia showed a 2.8-fold increase over the 10 years, and the age-adjusted prevalence showed a similar increasing trend (**Fig. 2A-C**). In addition, the increasing trend of prevalence was steeper in the geriatric group aged >70 years (**Fig. 2D-F**).

Fig. 2. Prevalence of dysphagia (A) total, (B) male, (C) female, (D) total according to

age group, (E) male according to age group, (F) female according to age group.

Risk of dysphagia by possible underlying etiologies

In the sample for the cross-sectional analysis (n = 39795 in each group), there were no differences in age or sex between the dysphagia and non-dysphagia groups due to age and sex matching; however, a large number of individuals with dysphagia had low income and comorbidities compared to individuals without dysphagia (**S1 Table**). The risk of dysphagia was significantly higher in each common etiology of dysphagia, with adjustments for age, sex, low-income status, and comorbidities (**Table 1**).

Table 1. Odds ratio of dysphagia by possible etiologies.

	Number of total individuals	Number of individuals with dysphagia	% of dysphagia	Odds ratio (95% CI)	
				Crude	Model 1*
**Stroke**					
Yes	23604	19074	80.8	9.54 (9.07–10.02)	7.86 (5.76–6.68)
No	55986	20721	37.0	1 (reference)	1 (reference)
**ND**					
Yes	13814	11590	83.9	10.00 (9.38–10.66)	6.20 (5.76–6.68)
No	65776	28205	42.9	1 (reference)	1 (reference)
**Cancer**					
Yes	6373	4828	75.8	3.54 (3.33–3.76)	5.59 (5.17–6.06)
No	73217	34967	47.8	1 (reference)	1 (reference)
**COPD**					

Yes	7028	5179	73.7	3.25 (3.06–3.44)	2.94 (2.71–3.18)
No	72562	34616	47.7	1 (reference)	1 (reference)

All p-values were < 0.001.

*A logistic regression analysis adjusted for age, sex, low-income status, and comorbidities.

CI: Confidence interval, ND: Neurodegenerative disease, COPD: Chronic obstructive pulmonary disease

The risk of dysphagia among the sampled comorbidities is noted from greatest to less association as follows: stroke (odds ratio [OR]: 7.86, 95% confidence interval [CI]: 5.76–6.68); $p < 0.001$), ND (OR: 6.20, 95% CI: 5.76–6.68; $p < 0.001$), cancer (OR: 5.59, 95% CI: 5.17–6.06; $p < 0.001$), and COPD (OR: 2.94, 95% CI: 2.71–3.18; $p < 0.001$).

Mortality

The Kaplan-Meier plot presented in Fig. 3 demonstrates a positive correlation between dysphagia and the risk of mortality. In addition, it is shown that this mortality risk increases yearly, particularly after the first 2 years of diagnosis (**Fig. 3**).

Fig. 3. Kaplan–Meier curve for mortality according to dysphagia.

The mortality rate in the dysphagia group was 93.11 per 1000 person-years, and the risk of death due to dysphagia increased 3.12 times (hazard ratio: 3.12, 95% CI: 3.03–3.23) even when adjusted for age, sex, low-income status, and comorbidities (**Table 2**).

Table 2. Hazard ratio for mortality by dysphagia

Dysphagia	Number of individuals	Number of deaths	Duration (person-years)	Mortality rate (per 1000 person-years)	Hazard ratio (95% CI)	
					Crude	Model 1*
Yes	39192	18037	193721.19	93.11	3.25 (3.16–3.34)	3.12 (3.03–3.23)
No	39192	7192	266974.32	26.94	1 (reference)	1 (reference)

All *p*-values were < 0.001.

*A Cox proportional hazard regression analysis adjusted for age, sex, low income status, and comorbidities.

CI: Confidence interval

Discussion

In this study, it is shown that the overall incidence and prevalence of dysphagia increased with age. The increasing trend was conspicuous in the geriatric population, particularly those older than 70 years. To the best of our knowledge, this is the first report on the incidence, prevalence, and increasing trend of dysphagia in the entire adult Korean population. Most of the previously reported prevalence values of dysphagia came from questionnaire analyses or cross-sectional studies of small sample sizes, specific diseases, and specific populations [12–14,16–20]. The relationship between dysphagia and aging can first be explained by geriatric syndromes [7,8]. The swallowing function may decline with aging, followed by changes in swallowing physiology, such as weakness of the swallowing reflex, cough response, bolus propulsion, and increased residue [2,14,23–25]. The associated sarcopenia, frailty, pain,

inadequate dentition, and polypharmacy may even aggravate the decline in swallowing function [7–9,26,27]. Polypharmacy in particular includes sedative drugs, opioids, and also higher anticholinergic burden, which may impair cognitive and swallowing function [4]. Second, this finding further suggests that the life expectancy of the Korean population has been extended such that a sufficient number of older individuals have been registered for the investigation and follow-up of the incidence and prevalence of dysphagia in this study. The proportion of those aged over 65 years in Korea was 14% in 2017 and is expected to reach 25% by 2030 [28]. As aging is one of the causes of dysphagia, dysphagia in the geriatric population will increase further and will be a more common geriatric disorder that requires medical attention. Lastly, this finding implies that dysphagia may place a significant socioeconomic and medical burden on the older population. The Korean national statistics reported that medical costs for those older than 65 years were about 40% of all medical costs in 2019 and that cardiovascular disease, neoplasm, respiratory disease, and nervous system disorders were the main causes of medical expenditures [29]. The current study demonstrates that stroke, cancer, COPD, and ND significantly increase the risk of dysphagia and that the presence of dysphagia significantly increases mortality. Therefore, dysphagia in older individuals should also be considered an important cause of socioeconomic and medical burden, demanding more substantial resources and adequate screening, diagnosis, and management independently of the underlying comorbidities because in older adults the most prevalent type of dysphagia is oropharyngeal cause. Additionally, dysphagia burden may be highlighted because this analysis was conducted using the NHIS claim codes, including objectively defined dysphagia and etiologies, which were related to medical expenditure.

As identified in the cross-sectional analysis in this study, the presence of stroke, ND, cancer, and COPD was strongly associated with a high risk of dysphagia. This finding is consistent with that of previous studies [12,16–20], and the pathophysiology of dysphagia in each disease can be explained as follows. A more severe stroke would initially have more severe neurological deficits possibly, including dysphagia [30,31]. The lesion locations are also related to swallowing dysfunction. Cerebellar and brain stem lesions may impair swallowing physiology, and cerebral lesions may impair the oral phase, including mastication and bolus transport. Cortical lesions may impair orofacial motor control and pharyngeal peristalsis [16]. Moreover, cognitive deficits may impair the control of swallowing [16,32]. Although the pathophysiology of dysphagia in Parkinson's disease is not clearly identified [33], it is believed that striatal dopaminergic deficiency and Lewy bodies affect swallowing centers and that advanced stages and prolonged disease duration are associated with severe dysphagia [34]. In dementia, the mechanisms of dysphagia depend on the type and progression of dementia. Given the nature of dementia, the impairment of food recognition, swallowing control, and cough response due to cognitive deficits may be related to the aggravation of dysphagia [17]. In cancers, dysphagia is related to the tumor site, advanced stage, and treatment modalities. Cancers, particularly of the head and neck and upper gastrointestinal tract, may interfere with food passage, and treatment modalities such as tumor resection, chemotherapy, and radiotherapy may cause anatomical or neurological damage to swallowing function [35–37]. The increasing number of geriatric cancer survivors [38] is also assumed to increase the number of dysphagia cases even the mechanical cause of dysphagia is not the most common in older people. In COPD, respiratory-swallowing discoordination, tachypnea and/or

dyspnea, oropharyngeal swallowing dysfunction, and physical and emotional distress are associated with an increased risk of dysphagia [12,22,39,40]. In addition, this study simultaneously analyzed the relationship between dysphagia and various etiologies, including diseases and age factors, differentiating it from previous studies. Considering that dysphagia risk increases with comorbidity and that the increase in dysphagia is prominent in the geriatric population, it additionally indicates that the geriatric population are inevitably more susceptible to comorbidities and are unable to compensate for disease-related swallowing dysfunction [41].

The current study showed a relatively low prevalence of dysphagia compared to published studies. Our study showed that the crude prevalence of dysphagia ranged from 0.09% to 0.25%. Previous studies reported that the prevalence of dysphagia ranged from 16% to 22% in people older than 50 years [5,11], 11.4% to 40% in geriatric population older than 65 years of age living in the community, and 25.5% to 60% in hospital/ institutional settings [4,5,7,11–14]. This discrepancy is probably due to our operational definition, which included patients with dysphagia who received medical attention such as outpatient or inpatient physician care, instrumental swallowing tests, and swallowing therapies. Since the incidence and prevalence reported in this study are based on the records of claims, the current study excluded people with dysphagia who have not received medical services. Although physician care, instrumental swallowing tests, and swallowing therapies help evaluate dysphagia accurately, they have disadvantages of cost and time compared to self-reports or standardized swallowing assessments [9]. In addition, people with mild or transient dysphagia may have recovered without seeing a doctor or receiving medical care, thus could not be included

in the dysphagia group. Consequently, the relatively low prevalence of dysphagia in the current study indicates more potentially unmet medical services than expected in the population with dysphagia. In addition, the lower prevalence of dysphagia in our study compared to that in previous studies is because this study was conducted in the general population of Korea. It is known that the prevalence of dysphagia is higher in hospital/institution settings than in community dwellings [42,43].

The current study shows that dysphagia significantly increases long-term mortality. This finding is similar to the previous literature, which suggested that mortality due to dysphagia was associated with aspiration or asphyxia, regardless of the underlying cause [2,32,44]. Dysphagia fatality is additionally supported by previous literature, which reported that dysphagia could cause numerous complications, including malnutrition and dehydration, having a great impact on morbimortality, re-hospitalization, frailty, and quality of life [7,17,22,30,32,34,45]. As dysphagia can place a significant socioeconomic and medical burden on the Korean population, a careful analysis of the medical background is necessary to properly evaluate and treat dysphagia requiring medical attention including the review of potentially causative diseases and medications.

This study has several strengths and limitations. First, the absolute numerical results in this study should be interpreted with caution because of the specificity and sensitivity of the operational definition. To minimize over- or underestimation, we tried to make an objective operational definition of dysphagia using the codes from the NHIS database and to specify dysphagia, which incurs medical expenses using claim codes of instrumental swallowing tests, swallowing therapies, and percutaneous

gastrostomies. VFSS is considered the gold standard for identifying dysphagia [46,47] and is routinely performed in South Korea. Therefore, we intended to increase the sensitivity and specificity of detecting dysphagia by including people who received VFSS or FEES at least twice within 3 months. When a penetration or aspiration is detected by the instrumental swallowing tests, the follow-up tests is usually performed within 3 months to examine the recovery or deterioration of swallowing [48,49]. Conversely, people with preserved swallowing function are not followed up unless a sudden worsening of dysphagia occurs. We also included people who received multiple swallowing therapies within 1 month. If penetration and/or aspiration are detected by the instrumental swallowing tests, most people receive swallowing therapies at least once a week. In addition, the criterion of nasogastric tube insertions more than two times was used to define dysphagia in the previous study conducted in Taiwan [50]. People who underwent the percutaneous gastrostomy were included because most of the procedures are performed in patients with dysphagia [51]. This strict definition of dysphagia can lead to an underestimation of dysphagia due to the exclusion of subjective dysphagia and people with dysphagia but limited access to dysphagia evaluation and treatment. The possibility that dysphagia requiring medical attention was not captured due to limited access to medical services warrants further study to investigate this gap. However, this study is valuable because we could define objective dysphagia requiring medical attention, which is more directly associated with medical costs. Moreover, the current study used a long-term and large database to study the prevalence, incidence, and mortality of dysphagia at the general Korean population level. Although not a novel result, the current study provided robust evidence that the prevalence and incidence of dysphagia increase with age, aging and/or comorbidities

multiply the risk of dysphagia, and the presence of dysphagia significantly increases mortality.

Second, a few factors might undermine the representativeness of the current study. The operational definition possibly included more oropharyngeal than esophageal dysphagia because the instrumental swallowing tests (VFSS and FEES) and the therapies were usually aimed at evaluating and improving oropharyngeal dysphagia. In addition, the exclusion of the pediatric population and related congenital diseases might have influenced the analysis of the prevalence, incidence, and mortality of dysphagia in the current study.

Third, the types and severity of dysphagia and their relationship with aging and comorbidities were not reflected. Since people who received instrumental swallowing tests and/or percutaneous gastrostomy would already have a high risk of dysphagia and more severe deficits [52], it is likely that apparent but severe dysphagia would be registered to satisfy the operational definition of dysphagia.

Fourth, only four diseases were selected as medical conditions that could be related to dysphagia, and the causal relationship between those possible etiologies and dysphagia could not be evaluated by cross-sectional analysis. Future studies should consider more diverse diseases, such as pneumonia and frailty, as possible etiologies of dysphagia.

Lastly, it is difficult to extrapolate the results from this study to the entire world because medical and epidemiologic circumstances of dysphagia may be different in various countries. Nevertheless, it is assumed that similar trends of incidence,

prevalence, and mortality of dysphagia may be seen in aging population in developed countries where similar evaluation and treatment of dysphagia are being performed. In this regard, future studies on dysphagia using global data from various countries is necessary.

Conclusion

The incidence and prevalence of dysphagia cases requiring medical attention are increasing yearly, and dysphagia increases long-term mortality. The presence of stroke, ND, cancer, and COPD is associated with a high risk of dysphagia. As many countries are entering an aging or super-aging society, dysphagia will be a more common problem that requires appropriate interventions in daily clinical practice such as the rest of geriatrics syndromes.

Acknowledgements

This study used National Health Information Database (NHIS-2018-1-331) by National Health Insurance Service (NHIS). The author(s) declare no conflict of interest with NHIS and would like to thank the NHIS for the cooperation.

Competing interests: None

References

1. World Health Organization. International classification of functioning, disability and health (ICF); 2001.
2. Ebihara S, Sekiya H, Miyagi M, Ebihara T, Okazaki T. Dysphagia, dystussia, and aspiration pneumonia in elderly people. J Thorac Dis. 2016;8: 632-639. doi: 10.21037/jtd.2016.02.60.
3. Rommel N, Hamdy S. Oropharyngeal dysphagia: manifestations and diagnosis. Nat Rev Gastroenterol Hepatol. 2016;13: 49-59. doi: 10.1038/nrgastro.2015.199.
4. Castejón-Hernández S, Latorre-Vallbona N, Molist-Brunet N, Cubí-Montanyà D, Espaulella-Panicot J. Association between anticholinergic burden and oropharyngeal dysphagia among hospitalized older adults. Aging Clin Exp Res. 2021;33: 1981-1985. doi: 10.1007/s40520-020-01707-9.
5. Cook IJ, Kahrilas PJ. AGA technical review on management of oropharyngeal dysphagia. Gastroenterology. 1999;116: 455-478. doi: 10.1016/s0016-5085(99)70144-7.
6. Shamburek RD, Farrar JT. Disorders of the digestive system in the elderly. N Engl J Med. 1990;322: 438-443. doi: 10.1056/NEJM199002153220705.
7. Baijens LW, Clavé P, Cras P, Ekberg O, Forster A, Kolb GF, et al. European Society for Swallowing Disorders – European Union Geriatric Medicine Society white paper: oropharyngeal dysphagia as a geriatric syndrome. Clin Interv Aging. 2016;11: 1403-1428. doi: 10.2147/CIA.S107750.
8. Warnecke T, Dziewas R, Wirth R, Bauer JM, Prell T. Dysphagia from a neurogeriatric point of view: pathogenesis, diagnosis and management. Z Gerontol Geriatr. 2019;52: 330-335. doi: 10.1007/s00391-019-01563-x.

9. Cha S, Kim WS, Kim KW, Han JW, Jang HC, Lim S, et al. Sarcopenia is an independent risk factor for dysphagia in community-dwelling older adults. Dysphagia. 2019;34: 692-697. doi: 10.1007/s00455-018-09973-6.

10. Logemann JA. Effects of aging on the swallowing mechanism. Otolaryngol Clin North Am. 1990;23: 1045-1056. doi: 10.1016/s0030-6665(20)31188-9.

11. Roden DF, Altman KW. Causes of dysphagia among different age groups: a systematic review of the literature. Otolaryngol Clin North Am. 2013;46: 965-987. doi: 10.1016/j.otc.2013.08.008.

12. Roy N, Stemple J, Merrill RM, Thomas L. Dysphagia in the elderly: preliminary evidence of prevalence, risk factors, and socioemotional effects. Ann Otol Rhinol Laryngol. 2007;116: 858-865. doi: 10.1177/000348940711601112.

13. Kawashima K, Motohashi Y, Fujishima I. Prevalence of dysphagia among community-dwelling elderly individuals as estimated using a questionnaire for dysphagia screening. Dysphagia. 2004;19: 266-271. doi: 10.1007/s00455-004-0013-6.

14. Yang EJ, Kim MH, Lim JY, Paik NJ. Oropharyngeal Dysphagia in a community-based elderly cohort: the Korean longitudinal study on health and aging. J Korean Med Sci. 2013;28: 1534-1539. doi: 10.3346/jkms.2013.28.10.1534.

15. Doan TN, Ho WC, Wang LH, Chang FC, Nhu NT, Chou LW. Prevalence and methods for assessment of oropharyngeal dysphagia in older adults: A systematic review and meta-analysis. J Clin Med. 2022;11. doi: 10.3390/jcm11092605.

16. Martino R, Foley N, Bhogal S, Diamant N, Speechley M, Teasell R. Dysphagia after stroke: incidence, diagnosis, and pulmonary complications. Stroke. 2005;36: 2756-2763. doi: 10.1161/01.STR.0000190056.76543.eb.

17. Alagiakrishnan K, Bhanji RA, Kurian M. Evaluation and management of oropharyngeal dysphagia in different types of dementia: a systematic review. Arch Gerontol Geriatr. 2013;56: 1-9. doi: 10.1016/j.archger.2012.04.011.

18. Takizawa C, Gemmell E, Kenworthy J, Speyer R. A systematic review of the prevalence of oropharyngeal dysphagia in stroke, Parkinson's disease, Alzheimer's disease, head injury, and pneumonia. Dysphagia. 2016;31: 434-441. doi: 10.1007/s00455-016-9695-9.

19. Frowen J, Hughes R, Skeat J. The prevalence of patient-reported dysphagia and oral complications in cancer patients. Support Care Cancer. 2020;28: 1141-1150. doi: 10.1007/s00520-019-04921-y.

20. Mercadante S, Aielli F, Adile C, Ferrera P, Valle A, Fusco F, et al. Prevalence of oral mucositis, dry mouth, and dysphagia in advanced cancer patients. Support Care Cancer. 2015;23: 3249-3255. doi: 10.1007/s00520-015-2720-y.

21. Song SO, Jung CH, Song YD, Park CY, Kwon HS, Cha BS, et al. Background and data configuration process of a nationwide population-based study using the Korean national health insurance system. Diabetes Metab J. 2014;38: 395–403. doi: 10.4093/dmj.2014.38.5.395.

22. Lin TF, Shune S. Chronic obstructive pulmonary disease and dysphagia: A synergistic review. Geriatrics (Basel). 2020;5: 45. doi: 10.3390/geriatrics5030045.

23. Rofes L, Arreola V, Romea M, Palomera E, Almirall J, Cabré M, et al. Pathophysiology of oropharyngeal dysphagia in the frail elderly. Neurogastroenterol Motil. 2010;22: 851-858, e230. doi: 10.1111/j.1365-2982.2010.01521.x.

24. Logemann JA, Pauloski BR, Rademaker AW, Colangelo LA, Kahrilas PJ, Smith CH. Temporal and biomechanical characteristics of oropharyngeal swallow in younger

and older men. J Speech Lang Hear Res. 2000;43: 1264-1274. doi: 10.1044/jslhr.4305.1264.

25. Yoshikawa M, Yoshida M, Nagasaki T, Tanimoto K, Tsuga K, Akagawa Y, et al. Aspects of swallowing in healthy dentate elderly persons older than 80 years. J Gerontol A Biol Sci Med Sci. 2005;60: 506-509. doi: 10.1093/gerona/60.4.506.

26. Nishida T, Yamabe K, Honda S. Dysphagia is associated with oral, physical, cognitive and psychological frailty in Japanese community-dwelling elderly persons. Gerodontology. 2020;37: 185–190. doi: 10.1111/ger.12455.

27. Inui A, Takahashi I, Kurauchi S, Soma Y, Oyama T, Tamura Y, et al. Oral conditions and dysphagia in Japanese, community-dwelling middle- and older- aged adults, independent in daily living. Clin Interv Aging. 2017;12: 515-521. doi: 10.2147/CIA.S132637.

28. Korea National Statistical Office. Estimated Population by Age Group; 2019. [Cited 15 September 2022]. Available from: http://kostat.go.kr.

29. Korean National Health Insurance Service; 2019 National Health Insurance Statistical Yearbook. [Cited 15 September 2022]. Available from: https://www.hira.or.kr/bbsDummy.do?pgmid=HIRAA020045020000. Health Insurance Review & Assessment Service. p. 2020.

30. Arnold M, Liesirova K, Broeg-Morvay A, Meisterernst J, Schlager M, Mono ML, et al. Dysphagia in acute stroke: incidence, burden and impact on clinical outcome. PLOS ONE. 2016;11: e0148424. doi: 10.1371/journal.pone.0148424.

31. Paciaroni M, Mazzotta G, Corea F, Caso V, Venti M, Milia P, et al. Dysphagia following Stroke. Eur Neurol. 2004;51: 162-167. doi: 10.1159/000077663.

32. Feng MC, Lin YC, Chang YH, Chen CH, Chiang HC, Huang LC, et al. The

mortality and the risk of aspiration pneumonia related with dysphagia in stroke patients. J Stroke Cerebrovasc Dis. 2019;28: 1381-1387. doi: 10.1016/j.jstrokecerebrovasdis.2019.02.011.

33. Michou E, Hamdy S. Dysphagia in Parkinson's disease: a therapeutic challenge? Expert Rev Neurother. 2010;10: 875-878. doi: 10.1586/ern.10.60.

34. Suttrup I, Warnecke T. Dysphagia in Parkinson's disease. Dysphagia. 2016;31: 24-32. doi: 10.1007/s00455-015-9671-9.

35. Shune SE, Karnell LH, Karnell MP, Van Daele DJ, Funk GF. Association between severity of dysphagia and survival in patients with head and neck cancer. Head Neck. 2012;34: 776-784. doi: 10.1002/hed.21819.

36. Frowen J. Dysphagia in patients with non-head and neck cancer. Curr Opin Otolaryngol Head Neck Surg. 2019;27: 162-167. doi: 10.1097/MOO.0000000000000541.

37. Hutcheson KA, Nurgalieva Z, Zhao H, Gunn GB, Giordano SH, Bhayani MK, et al. Two-year prevalence of dysphagia and related outcomes in head and neck cancer survivors: an updated SEER-Medicare analysis. Head Neck. 2019;41: 479-487. doi: 10.1002/hed.25412.

38. Bray F, Ferlay J, Soerjomataram I, Siegel RL, Torre LA, Jemal A. Global cancer statistics 2018: GLOBOCAN estimates of incidence and mortality worldwide for 36 cancers in 185 countries. CA Cancer J Clin. 2018;68: 394-424. doi: 10.3322/caac.21492.

39. Good-Fratturelli MD, Curlee RF, Holle JL. Prevalence and nature of dysphagia in VA patients with COPD referred for videofluoroscopic swallow examination. J Commun Disord. 2000;33: 93-110. doi: 10.1016/s0021-9924(99)00026-x.

40. Robinson DJ, Jerrard-Dunne P, Greene Z, Lawson S, Lane S, O'Neill D.

Oropharyngeal dysphagia in exacerbations of chronic obstructive pulmonary disease. European Geriatric Medicine. 2011;2: 201-203. doi: 10.1016/j.eurger.2011.01.003.

41. Muhle P, Wirth R, Glahn J, Dziewas R. Age-related changes in swallowing. Physiology and pathophysiology. Nervenarzt. 2015;86: 440-451. doi: 10.1007/s00115-014-4183-7.

42. Clavé P, Shaker R. Dysphagia: current reality and scope of the problem. Nat Rev Gastroenterol Hepatol. 2015;12: 259-270. doi: 10.1038/nrgastro.2015.49.

43. Lin LC, Wu SC, Chen HS, Wang TG, Chen MY. Prevalence of impaired swallowing in institutionalized older people in Taiwan. J Am Geriatr Soc. 2002;50: 1118-1123. doi: 10.1046/j.1532-5415.2002.50270.x.

44. Marik PE, Kaplan D. Aspiration pneumonia and dysphagia in the elderly. Chest. 2003;124: 328-336. doi: 10.1378/chest.124.1.328.

45. Ortega O, Martín A, Clavé P. Diagnosis and management of oropharyngeal dysphagia among older persons, state of the art. J Am Med Dir Assoc. 2017;18: 576-582. doi: 10.1016/j.jamda.2017.02.015.

46. Palmer JB, Kuhlemeier KV, Tippett DC, Lynch C. A protocol for the videofluorographic swallowing study. Dysphagia. 1993;8: 209-214. doi: 10.1007/BF01354540.

47. Martin-Harris B, Jones B. The videofluorographic swallowing study. Phys Med Rehabil Clin N Am. 2008;19: 769-785. doi: 10.1016/j.pmr.2008.06.004.

48. Lee WH, Lim MH, Seo HG, Seong MY, Oh BM, Kim S. Development of a novel prognostic model to predict 6-month swallowing recovery after ischemic stroke. Stroke. 2020;51: 440–448. doi: 10.1161/STROKEAHA.119.027439.

49. Sienza K, Kim Y, Park T, Oh B. Recovery of laryngeal closure in post-stroke survivors. Clin Arch Commun Disord. 2018;3: 89–94. doi: 10.21849/cacd.2018.00332.

50. Ho CH, Lin WC, Hsu YF, Lee IH, Hung YC. One-year risk of pneumonia and mortality in patients with poststroke dysphagia: A nationwide population-based study. J Stroke Cerebrovasc Dis. 2018;27: 1311–1317. doi: 10.1016/j.jstrokecerebrovasdis.2017.12.017.

51. Rahnemai-Azar AA, Rahnemaiazar AA, Naghshizadian R, Kurtz A, Farkas DT. Percutaneous endoscopic gastrostomy: indications, technique, complications and management. World J Gastroenterol. 2014;20: 7739–7751. doi: 10.3748/wjg.v20.i24.7739.

52. González-Fernández M, Gardyn M, Wyckoff S, Ky PK, Palmer JB. Validation of ICD-9 Code 787.2 for identification of individuals with dysphagia from administrative databases. Dysphagia. 2009;24: 398-402. doi: 10.1007/s00455-009-9216-1.

Supporting information

S1 Table. Baseline characteristics according to dysphagia in the year of 2010.

Fig 1

Click here to access/download;Figure;Fig1_230301.tif

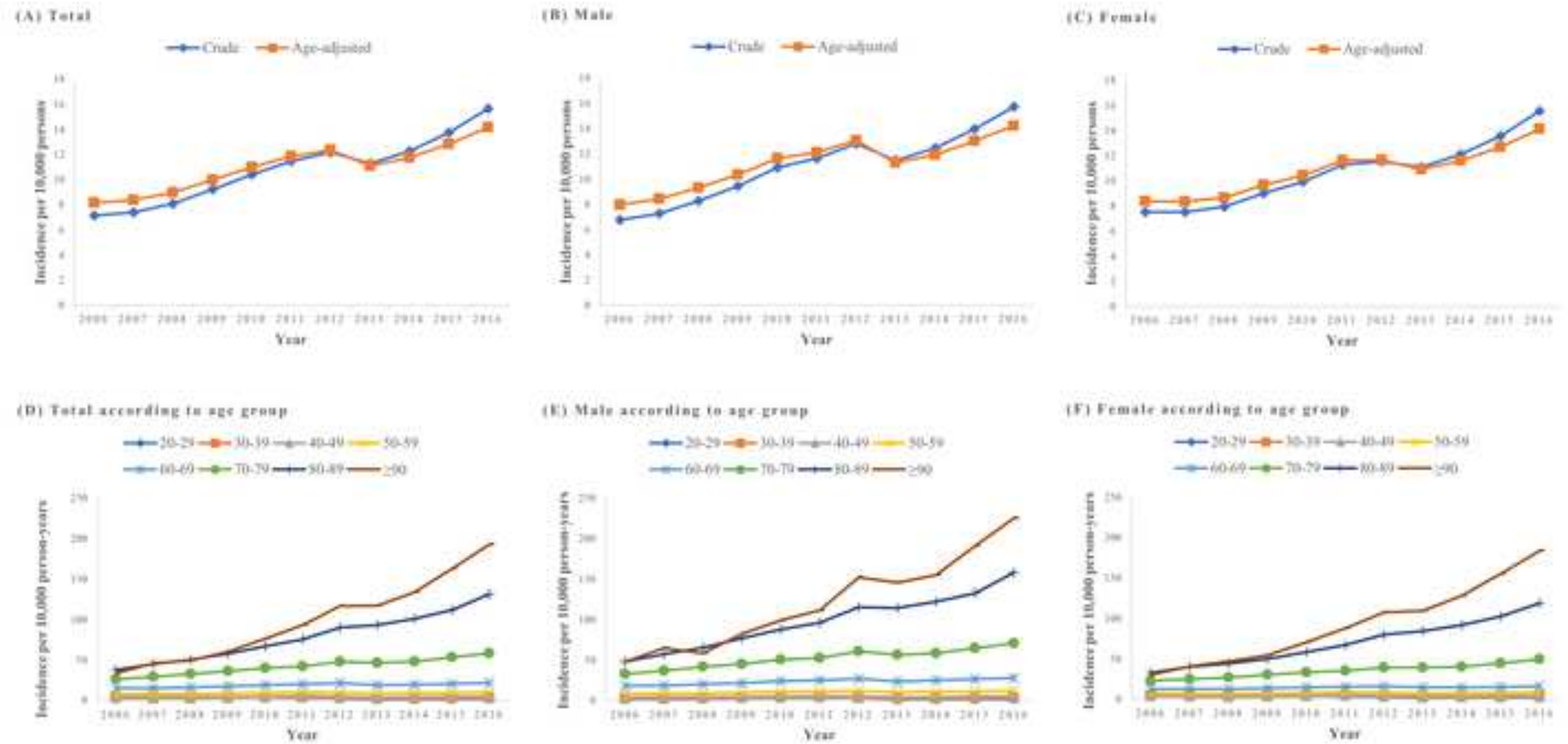

Fig 2

Click here to access/download;Figure;Fig2_230301.tif

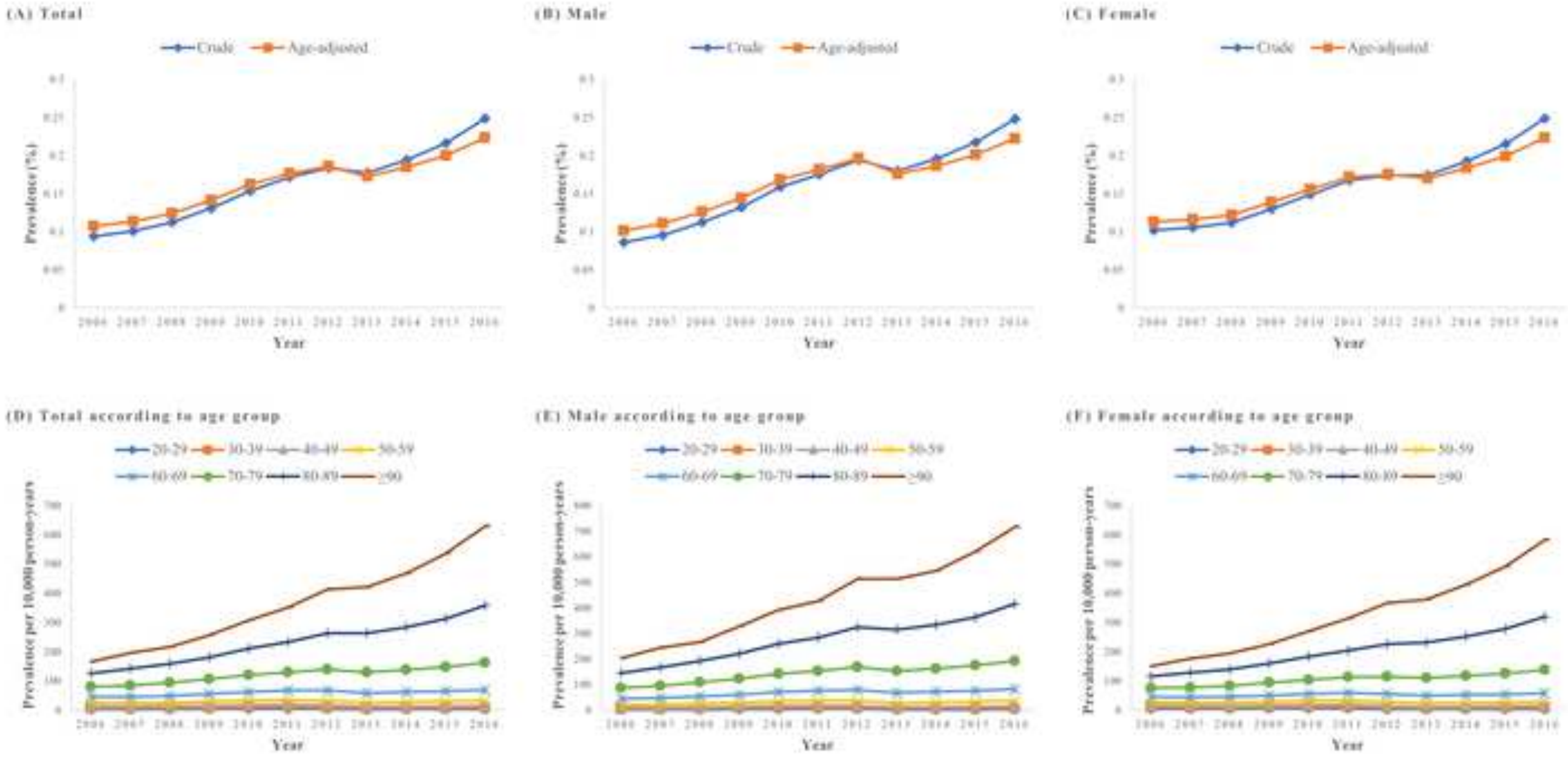

Click here to access/download;Figure;FIG3_22.12.tif

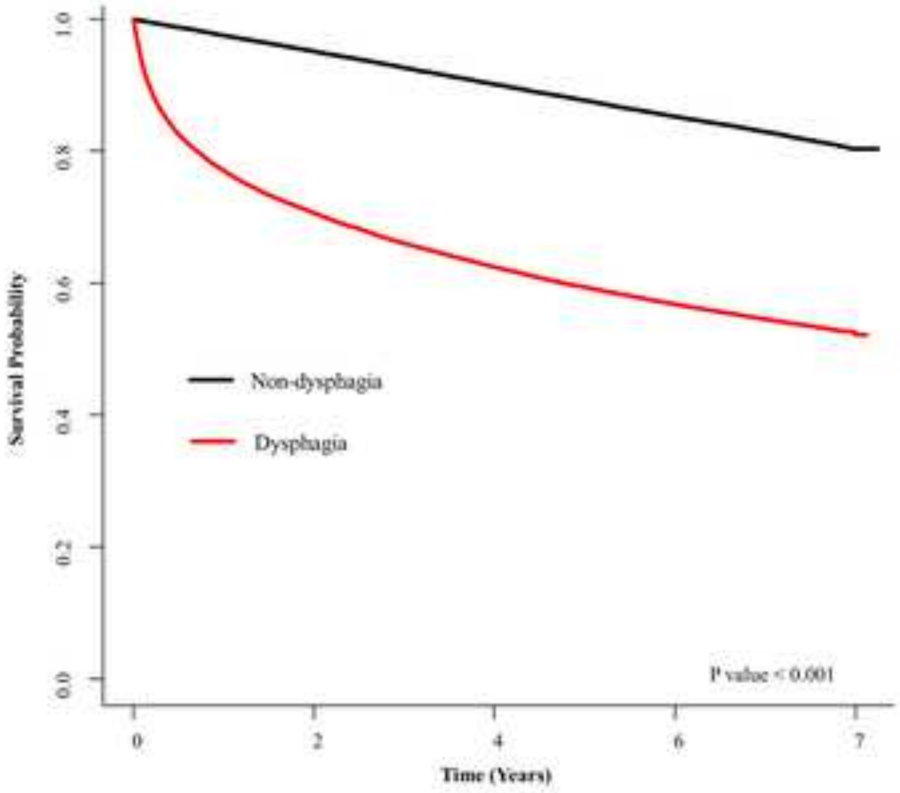

No. of survivors during follow-up					
Non-dysphagia	39192	37289	35331	33412	32511
Dysphagia	39192	27818	24652	22452	21563

Click here to access/download
Supporting Information
Supporting information.docx

Trends in the incidence and prevalence of dysphagia requiring medical attention among adults in South Korea, 2006–2016: A nationwide population study

Short title: Trends of dysphagia in South Korea

SuYeon Kwon[1], Seungwoo Cha[2], Junsik Kim[2], Kyungdo Han[3], Nam-Jong Paik[2], Won-Seok Kim[2*]

[1]Department of Rehabilitation Medicine, Ewha Woman's University Seoul Hospital, School of Medicine, Seoul, Republic of Korea

[2]Department of Rehabilitation Medicine, Seoul National University College of Medicine, Seoul National University Bundang Hospital, Gyeonggi-do, Republic of Korea

[3]Department of Statistics and Actuarial Science, Soongsil University, Seoul, Republic of Korea

***Corresponding author**

E-mail: wondol77@gmail.com (WSK)

Keywords: Dysphagia, Incidence, Prevalence, Mortality, Hazard Ratio

Abstract

The prevalence of dysphagia is increasing, resulting in socioeconomic burden, but previous reports have only been based on a specific population. Therefore, we aimed to investigate the nationwide incidence and prevalence of dysphagia requiring medical attention to provide adequate information for healthcare planning and resource allocation. In this nationwide retrospective cohort study, the data of adults aged ≥20 years recorded from 2006 to 2016 were sourced from the Korean National Health Insurance Service database. Medical claim codes based on ICD-10-CM were used to define dysphagia and possible causes. The annual incidence and prevalence of dysphagia were calculated. Cox regression was used to estimate dysphagia risk in people with possible dysphagia etiology. Survival analysis was performed to estimate the mortality and hazard ratio of dysphagia. The crude annual incidence of dysphagia increased continuously from 7.14 in 2006 to 15.64 in 2016. The crude annual prevalence of dysphagia in 2006 was 0.09% and increased annually to 0.25% in 2016. Stroke (odds ratio [OR]: 7.86, 95% confidence interval [CI]: 5.76–6.68), neurodegenerative disease (OR: 6.20, 95% CI: 5.76–6.68), cancer (OR: 5.59, 95% CI: 5.17–6.06), and chronic obstructive pulmonary disease (OR: 2.94, 95% CI: 2.71–3.18) were associated with a high risk of dysphagia. The mortality in the dysphagia group was 3.12 times higher than that in the non-dysphagia group (hazard ratio: 3.12, 95% CI: 3.03–3.23). The incidence and prevalence of dysphagia requiring medical attention are increasing annually. The increasing trend was conspicuous in the geriatric group. The presence of stroke, neurodegenerative disease, cancer, and chronic obstructive pulmonary disease is associated with a high risk of dysphagia. Therefore, adequate

screening, diagnosis, and management of dysphagia in the older population must be emphasized in geriatric healthcare.

Introduction

Swallowing is the process of passing solid and liquid food from the oral cavity, pharynx, and esophagus into the stomach with appropriate coordination [1]. Normal physiological swallowing is important not only for adequate nutrition and hydration but also for prevention of complications, such as aspiration pneumonia and asphyxia [2,3]. The World Health Organization has defined dysphagia as the difficulty in forming or moving a bolus safely from the oral cavity to the esophagus [4] and has recognized it as a medical disability associated with increased morbidity, mortality, and cost of treatment [1,3].

The swallowing process can be disrupted by diverse diseases such as stroke, traumatic brain injury, neurodegenerative disease (ND), motor neuron diseases, ~~and~~ cancers [5,6]. Since these diseases are increasing with the aging of the population, the incidence of dysphagia, as one of the associated complications, is expected to increase accordingly [7,8]. In addition, aging is associated with dysphagia due to muscle wasting, delayed swallowing reflex, and neuromuscular incoordination [2,3,9,10]. Dysphagia is becoming more common in the geriatric population [8] as life expectancy increases. The prevalence of dysphagia in the geriatric population has been reported variably in different countries. In the United States, the prevalence of dysphagia in adults over 50 years of age ranges from 11.4% to 22% [5,11], and that in the geriatric population living in the community is 33% [12]. In Europe, the prevalence of dysphagia in the geriatric population living in the community ranges from 30% to 40%, and that in hospital/ institutional settings ranges from 25.5% to 60% [4,7]. In Asia, the prevalence of dysphagia in the geriatric population living in the community ranges from 13.8% to

33.7% [13,14]. A meta-analysis and systematic review of the multinational studies reported that dysphagia affects 30% of community-dwelling geriatric population, almost 50% of geriatric patients, and above 50% of nursing home residents [15]. Therefore, diagnosing and managing dysphagia is an important issue in geriatric healthcare.

The previously reported prevalence of dysphagia is not representative of the entire population because most of these reports have come from cross-sectional studies with small sample sizes, specific diseases (e.g., stroke, ND, cancer) [16–20], and specific healthcare settings (hospitalized/institution settings) with specific age groups (healthy or frail geriatric population) [12–14]. Hence, there is a lack of nationwide data on the prevalence, incidence, and mortality of dysphagia to establish relevant healthcare policies on the evaluation and management of dysphagia.

Therefore, the primary purpose of this study ~~was~~ to investigate the nationwide trend of the incidence and prevalence of dysphagia requiring medical attention from a large, representative, population-based cohort, using claim data from the Korean National Health Insurance Service (NHIS) database from 2006 to 2016. We further investigated the possible diseases associated with dysphagia, and the risk of long-term mortality in dysphagic individuals compared to non-dysphagic individuals.

Materials and Methods

Data source and study population

The data source for this study is the NHIS database of South Korea. The NHIS has granted access to researchers since 2014. The NHIS is a universal social health insurance that provides healthcare coverage and medical aid to most of the Korean population (97.2% and 2.8%, respectively) [21]. Therefore, the NHIS presumptively has all records of medical service claims and sociodemographic information of the entire Korean population. The claim codes used in the Korean NHIS are based on the International Classification of Disease-Tenth Revision, Clinical Modification (ICD-10-CM). The complete data of the adult population aged ≥20 years (inpatient and outpatient medical service claims and sociodemographic information) from 2006 to 2016 were extracted. This study was exempt from review by the Seoul National University Bundang Hospital Institutional Review Board (X-1807/483-904), complying with the requirements of the Declaration of Helsinki. Data from the Korean NHIS were fully anonymized for analyses, and the need for obtaining informed consent was waived.

Definition of dysphagia and diseases that may have caused dysphagia

To define dysphagia, diagnostic codes and medical claim codes related to diagnosis or treatment of dysphagia were used. If a person matched at least one of the following criteria, the individual was assumed to have dysphagia: 1) presence of the diagnostic code for dysphagia (R13), 2) swallowing therapies (MX141) at least twice within 1 month, 3) instrumental swallowing tests (E7011, E7012) such as videofluoroscopic swallowing studies (VFSS) or fiberoptic endoscopic evaluation of swallowing (FEES) at least twice within 3 months, 4) nasogastric tube insertions

(Q2621, Q2622) at least twice within 3 months, or 5) procedure code claims for percutaneous gastrostomy (M6730, Q2612) at least once. In summary, the operational definition tried to include dysphagic people who received medical attention for dysphagia diagnosis or management during admission or outpatient visits.

Medical claim codes based on ICD-10-CM were used to define the diseases that may have caused dysphagia: 1) stroke as diagnostic codes of I63 and I64 and brain imaging (computed tomography or magnetic resonance imaging) during admission, 2) ND as diagnostic codes claimed for Parkinson's disease (G20 and V124) or those claimed for dementia (F00-03, G30, G31, G231, G310, G318, and F107), 3) cancer as diagnostic code of C codes (C00-14, C15-25, C32-34, C50, C53, C54, C56, C61, C62, C64, C67, C70-72, C73, C81-86, C90-95) and V193, and 4) chronic obstructive pulmonary disease (COPD) as diagnostic codes of J41-J44. V193 and V194 are specific deductible insurance codes for cancer and Parkinson's disease respectively in Korea. These four diseases were selected because they have been associated with dysphagia in previous studies [18,19,22].

Other variables

Other epidemiologic factors including age, sex, and income, were obtained and stratified into quartiles for statistical analyses. Data regarding death from various causes were obtained from the NHIS database linked to Statistics Korea, which records deaths of Korean citizens.

Statistics

The crude annual incidence was calculated by dividing the number of new patients with dysphagia each year by 10,000 person-years at risk. People diagnosed with dysphagia before 2006 were excluded as they would not represent new cases. The age-adjusted incidence and the incidence of dysphagia in each age group were calculated for each year to control for different age distributions throughout the years. The crude annual prevalence was calculated by dividing the number of people with dysphagia each year by the total of 10,000 people. Individuals who did not meet the operational definition of dysphagia in that year were excluded, even though they might have met the definition in the previous years. The age-adjusted prevalence and prevalence of dysphagia in each age group were also calculated.

To investigate the risk of dysphagia in possible common etiologies of dysphagia and the risk of mortality in dysphagia, data of people who were defined as having dysphagia for the first time in 2010 (n = 39795) were extracted and these individuals were followed up until 2016; these data were compared with the data of the age- and sex-matched population without dysphagia (n = 39795). The chi-square test was used to compare categorical variables between the dysphagia and non-dysphagia groups. Logistic regression analysis was used to determine the risk of dysphagia in each possible common etiology of dysphagia (stroke, ND, cancer, and COPD) in 2010. Adjustments for age, sex, low-income status, and comorbidities were made. Multivariate adjusted Cox proportional hazard regression analysis was used to identify the risk of mortality in the dysphagia group. Time-to-event analysis was used to estimate the mortality rate, and a Kaplan–Meier survival curve was plotted and

compared using the log-rank test. All statistical analyses were performed using SAS version 9.4 (SAS Institute Inc., Cary, NC, USA). Statistical significance was set at P < 0.05.

Results

Incidence of dysphagia

The crude annual incidence of dysphagia increased every year from 7.14 in 2006 to 15.64 in 2016, and the age-adjusted annual incidence showed a similar increasing trend (**Fig. 1A-C**). In addition, the annual incidence of dysphagia increased more prominently in the geriatric group aged >70 years. (**Fig. 1D-F**).

Fig. 1. Incidence of dysphagia between 2006 and 2016 (A) total, (B) male, (C) female, (D) total according to age group, (E) male according to age group, (F) female according to age group.

Prevalence of dysphagia

The crude annual prevalence of dysphagia showed a 2.8-fold increase over the 10 years, and the age-adjusted prevalence showed a similar increasing trend (**Fig. 2A-C**). In addition, the increasing trend of prevalence was steeper in the geriatric group aged >70 years (**Fig. 2D-F**).

Fig. 2. Prevalence of dysphagia (A) total, (B) male, (C) female, (D) total according to

age group, (E) male according to age group, (F) female according to age group.

Risk of dysphagia by possible underlying etiologies

In the sample for the cross-sectional analysis (n = 39795 in each group), there were no differences in age or sex between the dysphagia and non-dysphagia groups due to age and sex matching; however, a large number of individuals with dysphagia had low income and comorbidities compared to individuals without dysphagia (**S1 Table**). The risk of dysphagia was significantly higher in each common etiology of dysphagia, with adjustments for age, sex, low-income status, and comorbidities (**Table 1**).

Table 1. Odds ratio of dysphagia by possible etiologies.

	Number of total individuals	Number of individuals with dysphagia	% of dysphagia	Odds ratio (95% CI)	
				Crude	Model 1*
**Stroke**					
Yes	23604	19074	80.8	9.54 (9.07–10.02)	7.86 (5.76–6.68)
No	55986	20721	37.0	1 (reference)	1 (reference)
**ND**					
Yes	13814	11590	83.9	10.00 (9.38–10.66)	6.20 (5.76–6.68)
No	65776	28205	42.9	1 (reference)	1 (reference)
**Cancer**					
Yes	6373	4828	75.8	3.54 (3.33–3.76)	5.59 (5.17–6.06)
No	73217	34967	47.8	1 (reference)	1 (reference)
**COPD**					

Yes	7028	5179	73.7	3.25 (3.06–3.44)	2.94 (2.71–3.18)
No	72562	34616	47.7	1 (reference)	1 (reference)

All *p*-values were < 0.001.

*A logistic regression analysis adjusted for age, sex, low-income status, and comorbidities.

CI: Confidence interval, ND: Neurodegenerative disease, COPD: Chronic obstructive pulmonary disease

The risk of dysphagia among the sampled comorbidities is noted from greatest to less association as follows: stroke (odds ratio [OR]: 7.86, 95% confidence interval [CI]: 5.76–6.68); p < 0.001), ND (OR: 6.20, 95% CI: 5.76–6.68; p < 0.001), cancer (OR: 5.59, 95% CI: 5.17–6.06; p < 0.001), and COPD (OR: 2.94, 95% CI: 2.71–3.18; p < 0.001).

Mortality

The Kaplan-Meier plot presented in Fig. 3 demonstrates a positive correlation between dysphagia and the risk of mortality. In addition, it is shown that this mortality risk increases yearly, particularly after the first 2 years of diagnosis (**Fig. 3**).

Fig. 3. Kaplan–Meier curve for mortality according to dysphagia.

The mortality rate in the dysphagia group was 93.11 per 1000 person-years, and the risk of death due to dysphagia increased 3.12 times (hazard ratio: 3.12, 95% CI: 3.03–3.23) even when adjusted for age, sex, low-income status, and comorbidities (**Table 2**).

Table 2. Hazard ratio for mortality by dysphagia

Dysphagia	Number of individuals	Number of deaths	Duration (person-years)	Mortality rate (per 1000 person-years)	Hazard ratio (95% CI)	
					Crude	Model 1*
Yes	39192	18037	193721.19	93.11	3.25 (3.16–3.34)	3.12 (3.03–3.23)
No	39192	7192	266974.32	26.94	1 (reference)	1 (reference)

All *p*-values were < 0.001.

*A Cox proportional hazard regression analysis adjusted for age, sex, low income status, and comorbidities.

CI: Confidence interval

Discussion

In this study, it is shown that the overall incidence and prevalence of dysphagia increased with age. The increasing trend was conspicuous in the geriatric population, particularly those older than 70 years. To the best of our knowledge, this is the first report on the incidence, prevalence, and increasing trend of dysphagia in the entire adult Korean population. Most of the previously reported prevalence values of dysphagia came from questionnaire analyses or cross-sectional studies of small sample sizes, specific diseases, and specific populations [12–14,16–20]. The relationship between dysphagia and aging can first be explained by geriatric syndromes [7,8]. The swallowing function may decline with aging, followed by changes in swallowing physiology, such as weakness of the swallowing reflex, cough response, bolus propulsion, and increased residue [2,14,23–25]. The associated sarcopenia, frailty, pain,

inadequate dentition, and polypharmacy may even aggravate the decline in swallowing function [7–9,26,27]. Polypharmacy in particular includes sedative drugs, opioids, and also higher anticholinergic burden, which may impair cognitive and swallowing function [4]. Second, this finding further suggests that the life expectancy of the Korean population has been extended such that a sufficient number of older individuals have been registered for the investigation and follow-up of the incidence and prevalence of dysphagia in this study. The proportion of those aged over 65 years in Korea was 14% in 2017 and is expected to reach 25% by 2030 [28]. As aging is one of the causes of dysphagia, dysphagia in the geriatric population will increase further and will be a more common geriatric disorder that requires medical attention. Lastly, this finding implies that dysphagia may place a significant socioeconomic and medical burden on the older population. The Korean national statistics reported that medical costs for those older than 65 years were about 40% of all medical costs in 2019 and that cardiovascular disease, neoplasm, respiratory disease, and nervous system disorders were the main causes of medical expenditures [29]. The current study demonstrates that stroke, cancer, COPD, and ND significantly increase the risk of dysphagia and that the presence of dysphagia significantly increases mortality. Therefore, dysphagia in older individuals should also be considered an important cause of socioeconomic and medical burden, demanding more substantial resources and adequate screening, diagnosis, and management independently of the underlying comorbidities because in older adults the most prevalent type of dysphagia is oropharyngeal cause. Additionally, dysphagia burden may be highlighted because this analysis was conducted using the NHIS claim codes, including objectively defined dysphagia and etiologies, which were related to medical expenditure.

As identified in the cross-sectional analysis in this study, the presence of stroke, ND, cancer, and COPD was strongly associated with a high risk of dysphagia. This finding is consistent with that of previous studies [12,16–20], and the pathophysiology of dysphagia in each disease can be explained as follows. A more severe stroke would initially have more severe neurological deficits possibly, including dysphagia [30,31]. The lesion locations are also related to swallowing dysfunction. Cerebellar and brain stem lesions may impair swallowing physiology, and cerebral lesions may impair the oral phase, including mastication and bolus transport. Cortical lesions may impair orofacial motor control and pharyngeal peristalsis [16]. Moreover, cognitive deficits may impair the control of swallowing [16,32]. Although the pathophysiology of dysphagia in Parkinson's disease is not clearly identified [33], it is believed that striatal dopaminergic deficiency and Lewy bodies affect swallowing centers and that advanced stages and prolonged disease duration are associated with severe dysphagia [34]. In dementia, the mechanisms of dysphagia depend on the type and progression of dementia. Given the nature of dementia, the impairment of food recognition, swallowing control, and cough response due to cognitive deficits may be related to the aggravation of dysphagia [17]. In cancers, dysphagia is related to the tumor site, advanced stage, and treatment modalities. Cancers, particularly of the head and neck and upper gastrointestinal tract, may interfere with food passage, and treatment modalities such as tumor resection, chemotherapy, and radiotherapy may cause anatomical or neurological damage to swallowing function [35–37]. The increasing number of geriatric cancer survivors [38] is also assumed to increase the number of dysphagia cases even the mechanical cause of dysphagia is not the most common in older people. In COPD, respiratory-swallowing discoordination, tachypnea and/or

dyspnea, oropharyngeal swallowing dysfunction, and physical and emotional distress are associated with an increased risk of dysphagia [12,22,39,40]. In addition, this study simultaneously analyzed the relationship between dysphagia and various etiologies, including diseases and age factors, differentiating it from previous studies. Considering that dysphagia risk increases with comorbidity and that the increase in dysphagia is prominent in the geriatric population, it additionally indicates that the geriatric population are inevitably more susceptible to comorbidities and are unable to compensate for disease-related swallowing dysfunction [41].

The current study showed a relatively low prevalence of dysphagia compared to published studies. Our study showed that the crude prevalence of dysphagia ranged from 0.09% to 0.25%. Previous studies reported that the prevalence of dysphagia ranged from 16% to 22% in people older than 50 years [5,11], 11.4% to 40% in geriatric population older than 65 years of age living in the community, and 25.5% to 60% in hospital/ institutional settings [4,5,7,11–14]. This discrepancy is probably due to our operational definition, which included patients with dysphagia who received medical attention such as outpatient or inpatient physician care, instrumental swallowing tests, and swallowing therapies. Since the incidence and prevalence reported in this study are based on the records of claims, the current study excluded people with dysphagia who have not received medical services. Although physician care, instrumental swallowing tests, and swallowing therapies help evaluate dysphagia accurately, they have disadvantages of cost and time compared to self-reports or standardized swallowing assessments [9]. In addition, people with mild or transient dysphagia may have recovered without seeing a doctor or receiving medical care, thus could not be included

in the dysphagia group. Consequently, the relatively low prevalence of dysphagia in the current study indicates more potentially unmet medical services than expected in the population with dysphagia. In addition, the lower prevalence of dysphagia in our study compared to that in previous studies is because this study was conducted in the general population of Korea. It is known that the prevalence of dysphagia is higher in hospital/institution settings than in community dwellings [42,43].

The current study shows that dysphagia significantly increases long-term mortality. This finding is similar to the previous literature, which suggested that mortality due to dysphagia was associated with aspiration or asphyxia, regardless of the underlying cause [2,32,44]. Dysphagia fatality is additionally supported by previous literature, which reported that dysphagia could cause numerous complications, including malnutrition and dehydration, having a great impact on morbimortality, re-hospitalization, frailty, and quality of life [7,17,22,30,32,34,45]. As dysphagia can place a significant socioeconomic and medical burden on the Korean population, a careful analysis of the medical background is necessary to properly evaluate and treat dysphagia requiring medical attention including the review of potentially causative diseases and medications.

This study has several strengths and limitations. First, the absolute numerical results in this study should be interpreted with caution because of the specificity and sensitivity of the operational definition. To minimize over- or underestimation, we tried to make an objective operational definition of dysphagia using the codes from the NHIS database and to specify dysphagia, which incurs medical expenses using claim codes of instrumental swallowing tests, swallowing therapies, and percutaneous

gastrostomies. VFSS is considered the gold standard for identifying dysphagia [46,47] and is routinely performed in South Korea. Therefore, we intended to increase the sensitivity and specificity of detecting dysphagia by including people who received VFSS or FEES at least twice within 3 months. When a penetration or aspiration is detected by the instrumental swallowing tests, the follow-up tests is usually performed within 3 months to examine the recovery or deterioration of swallowing [48,49]. Conversely, people with preserved swallowing function are not followed up unless a sudden worsening of dysphagia occurs. We also included people who received multiple swallowing therapies within 1 month. If penetration and/or aspiration are detected by the instrumental swallowing tests, most people receive swallowing therapies at least once a week. In addition, the criterion of nasogastric tube insertions more than two times was used to define dysphagia in the previous study conducted in Taiwan [50]. People who underwent the percutaneous gastrostomy were included because most of the procedures are performed in patients with dysphagia [51]. This strict definition of dysphagia can lead to an underestimation of dysphagia due to the exclusion of subjective dysphagia and people with dysphagia but limited access to dysphagia evaluation and treatment. The possibility that dysphagia requiring medical attention was not captured due to limited access to medical services warrants further study to investigate this gap. However, this study is valuable because we could define objective dysphagia requiring medical attention, which is more directly associated with medical costs. Moreover, the current study used a long-term and large database to study the prevalence, incidence, and mortality of dysphagia at the general Korean population level. Although not a novel result, the current study provided robust evidence that the prevalence and incidence of dysphagia increase with age, aging and/or comorbidities

multiply the risk of dysphagia, and the presence of dysphagia significantly increases mortality.

Second, a few factors might undermine the representativeness of the current study. The operational definition possibly included more oropharyngeal than esophageal dysphagia because the instrumental swallowing tests (VFSS and FEES) and the therapies were usually aimed at evaluating and improving oropharyngeal dysphagia. In addition, the exclusion of the pediatric population and related congenital diseases might have influenced the analysis of the prevalence, incidence, and mortality of dysphagia in the current study.

Third, the types and severity of dysphagia and their relationship with aging and comorbidities were not reflected. Since people who received instrumental swallowing tests and/or percutaneous gastrostomy would already have a high risk of dysphagia and more severe deficits [52], it is likely that apparent but severe dysphagia would be registered to satisfy the operational definition of dysphagia.

Fourth, only four diseases were selected as medical conditions that could be related to dysphagia, and the causal relationship between those possible etiologies and dysphagia could not be evaluated by cross-sectional analysis. Future studies should consider more diverse diseases, such as pneumonia and frailty, as possible etiologies of dysphagia.

Lastly, it is difficult to extrapolate the results from this study to the entire world because medical and epidemiologic circumstances of dysphagia may be different in various countries. Nevertheless, it is assumed that similar trends of incidence,

prevalence, and mortality of dysphagia may be seen in aging population in developed countries where similar evaluation and treatment of dysphagia are being performed. In this regard, future studies on dysphagia using global data from various countries is necessary.

Conclusion

The incidence and prevalence of dysphagia cases requiring medical attention are increasing yearly, and dysphagia increases long-term mortality. The presence of stroke, ND, cancer, and COPD is associated with a high risk of dysphagia. As many countries are entering an aging or super-aging society, dysphagia will be a more common problem that requires appropriate interventions in daily clinical practice such as the rest of geriatrics syndromes.

Acknowledgements

This study used National Health Information Database (NHIS-2018-1-331) by National Health Insurance Service (NHIS). The author(s) declare no conflict of interest with NHIS and would like to thank the NHIS for the cooperation.

~~Funding~~

~~This work was supported by Seoul National University Bundang Hospital (SNUBH) Research Fund (No. 06-2018-140).~~

Competing interests: None

References

1. World Health Organization. International classification of functioning, disability and health (ICF); 2001.

2. Ebihara S, Sekiya H, Miyagi M, Ebihara T, Okazaki T. Dysphagia, dystussia, and aspiration pneumonia in elderly people. J Thorac Dis. 2016;8: 632-639. doi: 10.21037/jtd.2016.02.60.

3. Rommel N, Hamdy S. Oropharyngeal dysphagia: manifestations and diagnosis. Nat Rev Gastroenterol Hepatol. 2016;13: 49-59. doi: 10.1038/nrgastro.2015.199.

4. Castejón-Hernández S, Latorre-Vallbona N, Molist-Brunet N, Cubí-Montanyà D, Espaulella-Panicot J. Association between anticholinergic burden and oropharyngeal dysphagia among hospitalized older adults. Aging Clin Exp Res. 2021;33: 1981-1985. doi: 10.1007/s40520-020-01707-9.

5. Cook IJ, Kahrilas PJ. AGA technical review on management of oropharyngeal dysphagia. Gastroenterology. 1999;116: 455-478. doi: 10.1016/s0016-5085(99)70144-7.

6. Shamburek RD, Farrar JT. Disorders of the digestive system in the elderly. N Engl J Med. 1990;322: 438-443. doi: 10.1056/NEJM199002153220705.

7. Baijens LW, Clavé P, Cras P, Ekberg O, Forster A, Kolb GF, et al. European Society for Swallowing Disorders – European Union Geriatric Medicine Society white paper: oropharyngeal dysphagia as a geriatric syndrome. Clin Interv Aging. 2016;11: 1403-1428. doi: 10.2147/CIA.S107750.

8. Warnecke T, Dziewas R, Wirth R, Bauer JM, Prell T. Dysphagia from a neurogeriatric point of view: pathogenesis, diagnosis and management. Z Gerontol Geriatr. 2019;52: 330-335. doi: 10.1007/s00391-019-01563-x.

9. Cha S, Kim WS, Kim KW, Han JW, Jang HC, Lim S, et al. Sarcopenia is an independent risk factor for dysphagia in community-dwelling older adults. Dysphagia. 2019;34: 692-697. doi: 10.1007/s00455-018-09973-6.

10. Logemann JA. Effects of aging on the swallowing mechanism. Otolaryngol Clin North Am. 1990;23: 1045-1056. doi: 10.1016/s0030-6665(20)31188-9.

11. Roden DF, Altman KW. Causes of dysphagia among different age groups: a systematic review of the literature. Otolaryngol Clin North Am. 2013;46: 965-987. doi: 10.1016/j.otc.2013.08.008.

12. Roy N, Stemple J, Merrill RM, Thomas L. Dysphagia in the elderly: preliminary evidence of prevalence, risk factors, and socioemotional effects. Ann Otol Rhinol Laryngol. 2007;116: 858-865. doi: 10.1177/000348940711601112.

13. Kawashima K, Motohashi Y, Fujishima I. Prevalence of dysphagia among community-dwelling elderly individuals as estimated using a questionnaire for dysphagia screening. Dysphagia. 2004;19: 266-271. doi: 10.1007/s00455-004-0013-6.

14. Yang EJ, Kim MH, Lim JY, Paik NJ. Oropharyngeal Dysphagia in a community-based elderly cohort: the Korean longitudinal study on health and aging. J Korean Med Sci. 2013;28: 1534-1539. doi: 10.3346/jkms.2013.28.10.1534.

15. Doan TN, Ho WC, Wang LH, Chang FC, Nhu NT, Chou LW. Prevalence and methods for assessment of oropharyngeal dysphagia in older adults: A systematic review and meta-analysis. J Clin Med. 2022;11. doi: 10.3390/jcm11092605.

16. Martino R, Foley N, Bhogal S, Diamant N, Speechley M, Teasell R. Dysphagia after stroke: incidence, diagnosis, and pulmonary complications. Stroke. 2005;36: 2756-2763. doi: 10.1161/01.STR.0000190056.76543.eb.

17. Alagiakrishnan K, Bhanji RA, Kurian M. Evaluation and management of oropharyngeal dysphagia in different types of dementia: a systematic review. Arch Gerontol Geriatr. 2013;56: 1-9. doi: 10.1016/j.archger.2012.04.011.

18. Takizawa C, Gemmell E, Kenworthy J, Speyer R. A systematic review of the prevalence of oropharyngeal dysphagia in stroke, Parkinson's disease, Alzheimer's disease, head injury, and pneumonia. Dysphagia. 2016;31: 434-441. doi: 10.1007/s00455-016-9695-9.

19. Frowen J, Hughes R, Skeat J. The prevalence of patient-reported dysphagia and oral complications in cancer patients. Support Care Cancer. 2020;28: 1141-1150. doi: 10.1007/s00520-019-04921-y.

20. Mercadante S, Aielli F, Adile C, Ferrera P, Valle A, Fusco F, et al. Prevalence of oral mucositis, dry mouth, and dysphagia in advanced cancer patients. Support Care Cancer. 2015;23: 3249-3255. doi: 10.1007/s00520-015-2720-y.

21. Song SO, Jung CH, Song YD, Park CY, Kwon HS, Cha BS, et al. Background and data configuration process of a nationwide population-based study using the Korean national health insurance system. Diabetes Metab J. 2014;38: 395–403. doi: 10.4093/dmj.2014.38.5.395.

22. Lin TF, Shune S. Chronic obstructive pulmonary disease and dysphagia: A synergistic review. Geriatrics (Basel). 2020;5: 45. doi: 10.3390/geriatrics5030045.

23. Rofes L, Arreola V, Romea M, Palomera E, Almirall J, Cabré M, et al. Pathophysiology of oropharyngeal dysphagia in the frail elderly. Neurogastroenterol Motil. 2010;22: 851-858, e230. doi: 10.1111/j.1365-2982.2010.01521.x.

24. Logemann JA, Pauloski BR, Rademaker AW, Colangelo LA, Kahrilas PJ, Smith CH. Temporal and biomechanical characteristics of oropharyngeal swallow in younger

and older men. J Speech Lang Hear Res. 2000;43: 1264-1274. doi: 10.1044/jslhr.4305.1264.

25. Yoshikawa M, Yoshida M, Nagasaki T, Tanimoto K, Tsuga K, Akagawa Y, et al. Aspects of swallowing in healthy dentate elderly persons older than 80 years. J Gerontol A Biol Sci Med Sci. 2005;60: 506-509. doi: 10.1093/gerona/60.4.506.

26. Nishida T, Yamabe K, Honda S. Dysphagia is associated with oral, physical, cognitive and psychological frailty in Japanese community-dwelling elderly persons. Gerodontology. 2020;37: 185–190. doi: 10.1111/ger.12455.

27. Inui A, Takahashi I, Kurauchi S, Soma Y, Oyama T, Tamura Y, et al. Oral conditions and dysphagia in Japanese, community-dwelling middle- and older- aged adults, independent in daily living. Clin Interv Aging. 2017;12: 515-521. doi: 10.2147/CIA.S132637.

28. Korea National Statistical Office. Estimated Population by Age Group; 2019. [Cited 15 September 2022]. Available from: http://kostat.go.kr.

29. Korean National Health Insurance Service; 2019 National Health Insurance Statistical Yearbook. [Cited 15 September 2022]. Available from: https://www.hira.or.kr/bbsDummy.do?pgmid=HIRAA020045020000. Health Insurance Review & Assessment Service. p. 2020.

30. Arnold M, Liesirova K, Broeg-Morvay A, Meisterernst J, Schlager M, Mono ML, et al. Dysphagia in acute stroke: incidence, burden and impact on clinical outcome. PLOS ONE. 2016;11: e0148424. doi: 10.1371/journal.pone.0148424.

31. Paciaroni M, Mazzotta G, Corea F, Caso V, Venti M, Milia P, et al. Dysphagia following Stroke. Eur Neurol. 2004;51: 162-167. doi: 10.1159/000077663.

32. Feng MC, Lin YC, Chang YH, Chen CH, Chiang HC, Huang LC, et al. The

mortality and the risk of aspiration pneumonia related with dysphagia in stroke patients. J Stroke Cerebrovasc Dis. 2019;28: 1381-1387. doi: 10.1016/j.jstrokecerebrovasdis.2019.02.011.

33. Michou E, Hamdy S. Dysphagia in Parkinson's disease: a therapeutic challenge? Expert Rev Neurother. 2010;10: 875-878. doi: 10.1586/ern.10.60.

34. Suttrup I, Warnecke T. Dysphagia in Parkinson's disease. Dysphagia. 2016;31: 24-32. doi: 10.1007/s00455-015-9671-9.

35. Shune SE, Karnell LH, Karnell MP, Van Daele DJ, Funk GF. Association between severity of dysphagia and survival in patients with head and neck cancer. Head Neck. 2012;34: 776-784. doi: 10.1002/hed.21819.

36. Frowen J. Dysphagia in patients with non-head and neck cancer. Curr Opin Otolaryngol Head Neck Surg. 2019;27: 162-167. doi: 10.1097/MOO.0000000000000541.

37. Hutcheson KA, Nurgalieva Z, Zhao H, Gunn GB, Giordano SH, Bhayani MK, et al. Two-year prevalence of dysphagia and related outcomes in head and neck cancer survivors: an updated SEER-Medicare analysis. Head Neck. 2019;41: 479-487. doi: 10.1002/hed.25412.

38. Bray F, Ferlay J, Soerjomataram I, Siegel RL, Torre LA, Jemal A. Global cancer statistics 2018: GLOBOCAN estimates of incidence and mortality worldwide for 36 cancers in 185 countries. CA Cancer J Clin. 2018;68: 394-424. doi: 10.3322/caac.21492.

39. Good-Fratturelli MD, Curlee RF, Holle JL. Prevalence and nature of dysphagia in VA patients with COPD referred for videofluoroscopic swallow examination. J Commun Disord. 2000;33: 93-110. doi: 10.1016/s0021-9924(99)00026-x.

40. Robinson DJ, Jerrard-Dunne P, Greene Z, Lawson S, Lane S, O'Neill D.

Oropharyngeal dysphagia in exacerbations of chronic obstructive pulmonary disease. European Geriatric Medicine. 2011;2: 201-203. doi: 10.1016/j.eurger.2011.01.003.

41. Muhle P, Wirth R, Glahn J, Dziewas R. Age-related changes in swallowing. Physiology and pathophysiology. Nervenarzt. 2015;86: 440-451. doi: 10.1007/s00115-014-4183-7.

42. Clavé P, Shaker R. Dysphagia: current reality and scope of the problem. Nat Rev Gastroenterol Hepatol. 2015;12: 259-270. doi: 10.1038/nrgastro.2015.49.

43. Lin LC, Wu SC, Chen HS, Wang TG, Chen MY. Prevalence of impaired swallowing in institutionalized older people in Taiwan. J Am Geriatr Soc. 2002;50: 1118-1123. doi: 10.1046/j.1532-5415.2002.50270.x.

44. Marik PE, Kaplan D. Aspiration pneumonia and dysphagia in the elderly. Chest. 2003;124: 328-336. doi: 10.1378/chest.124.1.328.

45. Ortega O, Martín A, Clavé P. Diagnosis and management of oropharyngeal dysphagia among older persons, state of the art. J Am Med Dir Assoc. 2017;18: 576-582. doi: 10.1016/j.jamda.2017.02.015.

46. Palmer JB, Kuhlemeier KV, Tippett DC, Lynch C. A protocol for the videofluorographic swallowing study. Dysphagia. 1993;8: 209-214. doi: 10.1007/BF01354540.

47. Martin-Harris B, Jones B. The videofluorographic swallowing study. Phys Med Rehabil Clin N Am. 2008;19: 769-785. doi: 10.1016/j.pmr.2008.06.004.

48. Lee WH, Lim MH, Seo HG, Seong MY, Oh BM, Kim S. Development of a novel prognostic model to predict 6-month swallowing recovery after ischemic stroke. Stroke. 2020;51: 440–448. doi: 10.1161/STROKEAHA.119.027439.

49. Sienza K, Kim Y, Park T, Oh B. Recovery of laryngeal closure in post-stroke survivors. Clin Arch Commun Disord. 2018;3: 89–94. doi: 10.21849/cacd.2018.00332.

50. Ho CH, Lin WC, Hsu YF, Lee IH, Hung YC. One-year risk of pneumonia and mortality in patients with poststroke dysphagia: A nationwide population-based study. J Stroke Cerebrovasc Dis. 2018;27: 1311–1317. doi: 10.1016/j.jstrokecerebrovasdis.2017.12.017.

51. Rahnemai-Azar AA, Rahnemaiazar AA, Naghshizadian R, Kurtz A, Farkas DT. Percutaneous endoscopic gastrostomy: indications, technique, complications and management. World J Gastroenterol. 2014;20: 7739–7751. doi: 10.3748/wjg.v20.i24.7739.

52. González-Fernández M, Gardyn M, Wyckoff S, Ky PK, Palmer JB. Validation of ICD-9 Code 787.2 for identification of individuals with dysphagia from administrative databases. Dysphagia. 2009;24: 398-402. doi: 10.1007/s00455-009-9216-1.

Supporting information

S1 Table. Baseline characteristics according to dysphagia in the year of 2010.

March, 12, 2023

Sina Azadnajafabad

Academic Editors

PLOS ONE

Dear Editor:

We wish to re-submit the manuscript titled : "Trends in the incidence and prevalence of dysphagia requiring medical attention among adults in South Korea, 2006–2016: A nationwide population study". The manuscript ID is PONE-D-22-35490.

We are deeply grateful to you and the reviewers of *PLOS ONE* for spending valuable time reviewing our manuscript. The comments and issues raised by the reviewers helped us improve the quality of the manuscript. We have made efforts to improve the manuscript and address the reviewers' comments. You will find our detailed responses below and the revisions to the manuscript in red texts.

We hope that you will find the revised manuscript suitable for publication in *PLOS ONE* and look forward to receiving your response.

Sincerely,

Won-Seok Kim, MD, PhD.
Department of Rehabilitation Medicine, Seoul National University College of Medicine, Seoul National University Bundang Hospital, 166 Gumi-ro, Bundang-gu, Seongnam-si, Gyeonggi-do, 463-707, South Korea
Email: wondol77@gmail.com
Phone: +82-31-787-7737
Fax: +82-31-787-4051

Responses to reviewer comments

We greatly appreciate the efforts of the reviewers for their constructive comments in evaluating our manuscript. Each comment has been carefully considered and responses have been provided for each point. The revisions we made in the manuscript are written in red. The responses to the reviewers and changes in the revised manuscript are as follows.

Reviewer #1

General considerations:

Comment 1: I suggest including in the first paragraph of your introduction the definition of dysphagia. It is not described anywhere. Here you can cite this paper: *Castejón-Hernández, S., Latorre-Vallbona, N., Molist-Brunet, N., Cubí-Montanyà, D., & Espaulella-Panicot, J. (2020). Association between anticholinergic burden and oropharyngeal dysphagia among hospitalized older adults. Aging Clinical and Experimental Research, 33(7), 1981–1985. doi:10.1007/s40520-020-01707-9*

Response: We thank the reviewer for the valuable suggestion. We have added the reference and definition of dysphagia by WHO in the first paragraph of the introduction section. We also have added the citation recommended by you as Reference number 4.

"The World Health Organization has defined dysphagia as the difficulty in forming or moving a bolus safely from the oral cavity to the esophagus and has recognized it as a medical disability associated with increased morbidity, mortality, and cost of treatment." (Line 50-53, Page 4)

Reference section:

4. Castejón-Hernández, S., Latorre-Vallbona, N., Molist-Brunet, N., Cubí-Montanyà, D., & Espaulella-Panicot, J. (2020). Association between anticholinergic burden and oropharyngeal dysphagia among hospitalized older adults. Aging Clinical and Experimental Research, 33(7), 1981–1985. doi:10.1007/s40520-020-01707-9.(Line 388-391, Page 21)

Comment 2: Please, do not use elderly to describe a population of older people. It is an ageist term. I suggest try to avoid language that might be deemed unacceptable or inappropriate (e.g. 'older people' is preferred to 'the elderly', the word 'senile' is best avoided). Take care with wording that might cause offence to ethnic or cultural groups"

Response: Thank you for pointing out the error in using the term "elderly". Based on your and reviewer #2's comments, we changed the expressions to "geriatric group" throughout the manuscript.

Comment 3: Try to avoid repetitive terms as dysphagia in every paragraph. Maybe you can describe your population as *dysphagic* or *non-dysphagic*. Even, you can use pronouns to refer to dysphagia in the same sentence.

Response: Thank you for the valuable comment. We changed the expressions throughout the manuscript.

Comment 4: Maybe, it could be more interesting to describe whole/general prevalence more than a national one, because although Korea is a populous country, not all the countries have millions of population and these data could not be extrapolated into another countries.

Response: Thank you for raising this issue. There are currently no global data on worldwide prevalence of dysphagia. Previous literature shows country-wise data on the prevalence of dysphagia among specific populations or risk groups. Therefore, we have put the greatest importance in reporting nationwide data on the incidence, prevalence, and mortality of dysphagia in Korea, and in defining the dysphagia requiring medical attention (related to medical costs or interventions) based on claim codes. We added this content to the discussion section as one of limitations.

"Lastly, it is difficult to extrapolate the results from this study to the entire world because medical and epidemiologic circumstances of dysphagia may be different in various countries. Nevertheless, it is assumed that similar trends of incidence, prevalence, and mortality of dysphagia may be seen in aging population in developed countries where similar evaluation and treatment of dysphagia are being performed. In this regard, future studies on dysphagia using global data from various countries is necessary." (Line 356-362, Page 18-19)

Comment 5: I am a little confused with the main aim of this study so maybe you should me more exact. If your objective is to know the prevalence of dysphagia independently of the cause (including mechanical, oropharyngeal, neurological … causes), please be explicit. In my opinion, it is not so relevant to determinate if they require medical attention or not. You only want to know a rate: the prevalence (percentage) of people affected by dysphagia

Response: We thank the reviewer for bring up this point. In this study, we have emphasized on dysphagia requiring medical attention (related to medical interventions and costs) and analyzing its incidence, prevalence, and associations with mortality. As per the primary objective of our study, we have defined dysphagia according to the diagnostic and medical claim codes used in the Korean NHIS which is based on the ICD-10-CM. This is stated in the inclusion criteria under the material and methods section.

"To define dysphagia, diagnostic codes and medical claim codes related to diagnosis or treatment of dysphagia were used. If a person matched at least one of the following criteria, the individual was assumed to have dysphagia: 1) presence of the diagnostic code for dysphagia (R13), 2) swallowing therapies (MX141) at least twice within 1 month, 3) instrumental swallowing tests (E7011, E7012) such as videofluoroscopic swallowing studies (VFSS) or fiberoptic endoscopic evaluation of swallowing (FEES) at least twice within 3 months, 4) nasogastric tube insertions (Q2621, Q2622) at least twice within 3 months, or 5) procedure code claims for percutaneous gastrostomy (M6730, Q2612) at least once." (Line 103-111, Page 6,7)

Hence any person receiving medical attention for dysphagia were categorized into dysphagia group. Therefore, we think that the term "dysphagia requiring medical attention" is the best term for specifying the objective of the study. As you have pointed out, the primary purpose is to investigate the prevalence and incidence, but our auxiliary purpose is to know the risk of

dysphagia in the specific diseases which have been well-known etiologies for dysphagia and to investigate the risk of mortality due to dysphagia. We have reworded our aim and stated our primary and secondary purpose of this study at the end of the introduction section.

"Therefore, the primary purpose of this study was to investigate the nationwide trend of the incidence and prevalence of dysphagia requiring medical attention from a large, representative, population-based cohort, using claim data from the Korean National Health Insurance Service (NHIS) database from 2006 to 2016. We further investigated the possible diseases associated with dysphagia, and the risk of long-term mortality in dysphagic individuals compared to non-dysphagic individuals." (Line 79-84, Page 5)

Comment 6: I am not sure about if the insertion of nasogastric tube insertions should be considered as diagnostic criteria of dysphagia. What about surgical procedures? Or abdominal acute pain? Constipation?... They are clearly not related to dysphagia.

Response: Thank you for bringing up this point. Patients who have a nasogastric tube are very likely to be unable to take sufficient food orally thus indicating obvious dysphagia. [1,2] As you have suggested that the one-time NG tube insertion may be due to the transient problem, so we defined the dysphagia when multiple NG tube insertions are required. Study by Ho et al. on the association of pneumonia and mortality in patients with poststroke dysphagia, also used the same definition of dysphagia for NG tube, where ≥2 nasogastric intubations was classified as dysphagia.[3]

Comment 7: I understand neurological disorders and cancer as a causing dysphagia… but COPD maybe is not the most important/prevalent cause of it… Please, check this bibliography which seems to be more philosophic than scientific (the journal has not impact factor).

Response: Thank you for the valuable comment. Stroke, neurodegenerative disease, cancer, and COPD were selected because they have been associated with dysphagia in many previous studies. Moreover, COPD is known to cause dysphagia due its dyspnea, short of breath, and related respiratory-swallowing discoordination. This is described in the discussion. As you suggested we have added 2 more studies by Good-Fratturelli, et al (Ref No. 39)[4] and Robinson DJ,et al(Ref No. 40)[5] to support the our viewpoint more scientifically.

Introduction section:

"These four diseases were selected because they have been associated with dysphagia in previous studies." (Line 123-124, Page 7*)*

Discussion section:

"In COPD, respiratory-swallowing discoordination, tachypnea and/or dyspnea, oropharyngeal swallowing dysfunction, and physical and emotional distress are associated with an increased risk of dysphagia." (Line 266-268, Page 14,15)

<u>Specific considerations</u>:

Comment 8: I propose you to add at the end of line n. 211: *"and also some side-effects from sedative drugs, opioids and also higher anticholinergic burden".* Here you can cite Castejon et al.

Response: Thank you for your valuable input. We modified your suggestion a little bit for cohesiveness and added it to the text under Discussion section.

"The associated sarcopenia, frailty, pain, inadequate dentition, and polypharmacy may even aggravate the decline in swallowing function. *Polypharmacy in particular includes sedative drugs, opioids, and also higher anticholinergic burden, which may impair cognitive and swallowing function."* (Line 218-222, Page 12,13)

Comment 9: I suggest adding after "management" in line 228: "*independently of the underlying diagnosis, because in older adults the most prevalent type of dysphagia is oropharyngeal cause".*

Response: Thank you for this suggestion. Accordingly, we added it to the text in the Discussion Section.

*"*Therefore, dysphagia in older individuals should also be considered an important cause of socioeconomic and medical burden, demanding more substantial resources and adequate screening, diagnosis, and management *independently of the underlying comorbidities because in older adults the most prevalent type of dysphagia is oropharyngeal cause."* (Line 235-239, Page 13)

Comment 10: In line 252, after "of dysphagia cases" it could be added the following sentence: *"even the mechanical cause of dysphagia is not the most common/prevalent in older people".*

Response: Thank you for your input. We added it to the text in the Discussion Section.

*"*The increasing number of geriatric cancer survivors is also assumed to increase the number of dysphagia cases *even the mechanical cause of dysphagia is not the most common in older people."* (Line 263-266, Page 14)

Comment 11: If you cite the paper I recommended you before, you can include in line 266: hospital/institution setting is *"ranged from 25.5% to 55%".*

Response: Thank you for pointing it out. Based on your and reviewer 2' comments, we revised the text to describe the prevalence of dysphagia in different countries in the introduction and in the discussion section. We have added the citation recommended by you as Reference number 4.

Introduction Section:

"The prevalence of dysphagia in the geriatric population has been reported variably in different countries. In the United States, the prevalence of dysphagia in adults over 50 years of

age ranges from 11.4% to 22%, and that in the geriatric population living in the community is 33%. In Europe, the prevalence of dysphagia in the geriatric population living in the community ranges from 30% to 40%, and that in hospital/ institutional settings ranges from 25.5% to 60%. *In Asia, the prevalence of dysphagia in the geriatric population living in the community ranges from 13.8% to 33.7%. A meta-analysis and systematic review of the multinational studies reported that dysphagia affects 30% of community-dwelling geriatric population, almost 50% of geriatric patients, and above 50% of nursing home residents."* (Line 61-70, Page 4,5)

Discussion Section:

"The current study showed a relatively low prevalence of dysphagia compared to published studies. Our study showed that the crude prevalence of dysphagia ranged from 0.09% to 0.25%. Previous studies reported that the prevalence of dysphagia *ranged from 16% to 22% in people older than 50 years, 11.4% to 40% in geriatric population older than 65 years of age living in the community, and 25.5% to 60% in hospital/ institutional settings."* (Line 277-280, Page 15)

Comment 12: Consider to add at the end of line 289: *"including the review of potentially causing medications or their side effects, as sedation or xerostomia".*

Response: As per your suggestion, we have modified the sentence a little bit for cohesiveness and added it to the text under the Discussion section.

"As dysphagia can place a significant socioeconomic and medical burden on the Korean population, a careful analysis of the medical background is necessary to properly evaluate and treat dysphagia requiring medical attention *including the review of potentially causative diseases and medications."* (Line 302-306, Page 16)

Comment 13: Lines 306 and 307: Maybe they should be removed because of I explained before about the nasogastric tube.

Response: After careful consideration, we decided to retain the sentence and added the description of nasogastric tube insertion in the definition criteria of dysphagia based on previous literature in the Discussion section. Also see the response to **Comment 6.**

"In addition, the criterion of nasogastric tube insertions more than two times was used to define dysphagia in the previous study conducted in Taiwan.." (Line 323-324, Page 17)

Comment 14: Lines 343 and 343: I propose to substitute "As they are common geriatric diseases and" for *"As many countries are entering an aging or super-aging society, dysphagia will be a more common problem that requires appropriate interventions in daily clinical practice such as the rest of geriatrics syndromes".*

Response: Thank you for this comment. As you suggested, we revised the conclusion.

*“*The presence of stroke, ND, cancer, and COPD is associated with a high risk of dysphagia. *As many countries are entering an aging or super-aging society, dysphagia will be a more common problem that requires appropriate interventions in daily clinical practice such as the rest of geriatrics syndromes.”* (Line 366-370, Page 19)

Reviewer #2

<u>General considerations</u>: This article seems to represent a unique review of dysphagia based on the intended population. The article is easy to follow in its entirety. Several considerations for review/minor editing:

Response: Thank you for your positive words about our study. Your comments truly helped us to improve the quality of the manuscript.

<u>Specific considerations</u>

Comment 1: Line 22-24: perhaps these sentences can be combined: ex "The prevalence of dysphagia and the resulting socioeconomic burden are known to be increasing, here we..."

Response: We thank the reviewer for the suggestion, however after careful consideration, we decided to keep the two sentences separately. Combining two sentences may not reduce the number of words and that the final sentence might be too long to understand.

The prevalence of dysphagia is increasing, resulting in socioeconomic burden, but previous reports have only been based on a specific population. Therefore, we aimed to investigate the nationwide incidence and prevalence of dysphagia requiring medical attention to provide adequate information for healthcare planning and resource allocation. (Line 22-26, Page 2)

Comment 2: Line 41-42: "elderly group older than 60 years" can probably be replaced with "geriatric population" as geriatric is a term used more frequently in the paper overall

Response: We agree with the reviewer and have revised the expression and used "geriatric group" and "geriatric population" throughout the manuscript.

Comment 3:

1) Line 58: Perhaps "the cause of" should be "associated with" as there isn't necessarily a causal relationship demonstrated.

2) Line 59: "or" should be "and" ; "Indeed" is not needed.

Response: Thank you for pointing out the error in the text. As per your suggestion, we have revised the sentences in the introduction section.

*"*In addition, aging is *associated with* dysphagia due to muscle wasting, delayed swallowing reflex, *and* neuromuscular incoordination. *Dysphagia* is becoming more common in the *geriatric population* as life expectancy increases." *(*Line 58-59, Page 4*)*

Comment 4: Lines 61-65: Reviewing the sources here, would caution using statistics from other countries given the paper here is specifically a South Korean analysis.

Response: We thank the reviewer for raising this point. This study used data of South Korean population specifically. So, we revised the text as you suggested to clearly describe where the citations come from. Furthermore, we tried to clearly describe where the country-wise statistics come from (e.g., Europe, US,etc.) in the introduction section..

"Dysphagia is becoming more common in the geriatric population as life expectancy increases. The prevalence of dysphagia in the geriatric population has been reported variably in different countries. In the United States, the prevalence of dysphagia in adults over 50 years of age ranges from 11.4% to 22%, and that in the geriatric population living in the community is 33%. In Europe, the prevalence of dysphagia in the geriatric population living in the community ranges from 30 to 40%, and that in hospital/ institutional settings ranges from 25.5% to 60%. In Asia, the prevalence of dysphagia in the geriatric population living in the community ranges from 13.8% to 33.7%. A meta-analysis and systematic review of the multinational studies reported that dysphagia affects 30% of community-dwelling geriatric population, almost 50% of geriatric patients, and above 50% of nursing home residents." (Line 60-70, Page 4,5)

Comment 5: Line 66: "However" is not needed

Response: Thank you for bringing it to our notice. We have removed "However," and revised the text in the introduction section.

*"*The previously reported prevalence of dysphagia is not representative of the entire population because most of these reports have come from cross-sectional studies with small sample sizes, specific diseases (e.g., stroke, ND, cancer), and specific healthcare settings (hospitalized/institution settings) with specific age groups (healthy or frail *geriatric population)."* (Line 72-76, Page 5)

Comment 6:

1) Line 73: Consider adding "the" after of.

2) Line 74: Perhaps instead of "in a large, representative," can simply state "from a". It is assumed a population database has those characteristics.

Response: As per your suggestion, we have revised the text in the Introduction section.

"Therefore, *the primary purpose of this study was* to investigate the nationwide trend of *the* incidence and prevalence of dysphagia requiring medical attention *from* a large, representative, population-based cohort, using claim data from the Korean National Health Insurance Service (NHIS) database from 2006 to 2016." (Line 79-82, Page 5)

Comment 7:

1) Line 78: "Therefore" not needed.

2) Line 80: "Thus" not needed

Response: As per your suggestion, we deleted “Therefore” at the beginning of the sentence and “thus” in front of emphasizing. However, after careful consideration, we decided to remove this final sentence of the introduction, in order to present the study purpose concisely.

#Line 84-88, Page 5 (removed): *This study aimed to present clear nationwide evidence on the prevalence, incidence, and mortality of dysphagia in South Korea, emphasizing the health-related impact of dysphagia in an aging society to provide adequate information for healthcare planning and resource allocation.*

Comment 8:

1) Line 87: consider "universal" instead of "mandatory"; at least in the United States the term "universal healthcare" is used, I can't comment on Canada or Europe.

2) Line 89: consider adding an ambiguous word such as "presumptively" after NHIS; this article makes a point of highlighting the weakness of the reporting system in the discussion. It is doubtfully a perfect system.

Response: Thank you for bringing it to our notice. We have revised the text in the materials and methods section.

*“*The NHIS is a *universal* social health insurance that provides healthcare coverage and medical aid to most of the Korean population (97.2% and 2.8%, respectively). Therefore, the NHIS *presumptively* has all records of medical service claims and sociodemographic information of the entire Korean population*.”* (Line 89-93, Page 6)

Comment 9: Line 100 (the entire section): The parameters investigated were inclusive and well described.

Response: We would like to thank the reviewer for appreciating the criteria.

Comment 10: Lines 112-117: Would use the same formatting used earlier in the section: replace the period after dysphagia in line 113 with a colon (:), then use commas (,) instead of semicolons (;) before "2)" and "3)" and "and 4)".

Response: Thank you for bringing it to our notice. We have revised the paragraph according to your **comment 10** and **comment 11**.

*“*Medical claim codes based on ICD-10-CM were used to define the diseases that may have caused dysphagia: 1) stroke as diagnostic codes of I63 and I64 and brain imaging (computed tomography or magnetic resonance imaging) during admission, 2) ND as diagnostic codes claimed for Parkinson’s disease (G20 and V124) or those claimed for dementia (F00-03, G30, G31, G231, G310, G318, and F107), 3) cancer as diagnostic code of C codes *(C00-14, C15-25, C32-34, C50, C53, C54, C56, C61, C62, C64, C67, C70-72, C73, C81-86, C90-95*) and V193, and 4) chronic obstructive pulmonary disease (COPD) as diagnostic codes of J41-J44. V193 and V194 are specific deductible insurance codes for cancer and Parkinson's disease

respectively *in Korea.* These four diseases were selected because they have been associated with dysphagia in previous studies." (Line 114-124, Page 7)

Comment 11: Lines 113-114 - The context of the sentence sounds like something was edited out or was intended to be removed but was kept. Were there also codes used for specific brain imaging to identify stroke that were supposed to be given ICD-10 codes?

Response: Thank you for the raising this point. In line 115-116, we intended to specifically define ischemic or hemorrhagic stroke patients, whose diagnosis was confirmed with not only ICD-10-CM codes but also with brain lesions on imaging studies such as brain CT or MRI. In the previous study, defining stroke using ICD-10 codes alone was not valid, and defining stroke using ICD-10 codes and medical claims of brain CT or MRI was more valid for estimating the prevalence of stroke (Kim et al.).[6]

Comment 12: Line 116 - perhaps in this section "cancer" should be described more. It was in fact elaborated on in the discussion, but it might be more consistent to given an explanation here, as this is the definitions section of the article.

Response: We thank the reviewer to the suggestion. The C codes (C00.00-C96) from ICD-10-CM includes various types of neoplasm, and we chose specific codes among them: oral cavity and pharynx (C00–C14), digestive organs (C15-25), respiratory and intrathoracic organs (C32-34), skin (C43), breast (C50), genital organs (C53, C54, C56, C61, C62), urinary tract (C64, C67), central nervous system (C70–72), thyroid (C73), lymphoma (C81–86), multiple myeloma (C90), and leukemia (C91–95). And V193 and V194 are specific deductible insurance codes for cancer and Parkinson's disease respectively in Korea.

We added this explanation in the operational definition under the Material and methods section.

" Medical claim codes based on ICD-10-CM were used to define the diseases that may have caused dysphagia: 1) stroke as diagnostic codes of I63 and I64 and brain imaging (computed tomography or magnetic resonance imaging) during admission, 2) ND as diagnostic codes claimed for Parkinson's disease (G20 and V124) or those claimed for dementia (F00-03, G30, G31, G231, G310, G318, and F107), 3) cancer as diagnostic code of C codes *(C00-14, C15-25, C32-34, C50, C53, C54, C56, C61, C62, C64, C67, C70-72, C73, C81-86, C90-95)* and V193, and 4) chronic obstructive pulmonary disease (COPD) as diagnostic codes of J41-J44. *V193 and V194 are specific deductible insurance codes for cancer and Parkinson's disease respectively in Korea.* These four diseases were selected because they have been associated with dysphagia in previous studies." (Line 114-124, Page 7)

Comment 13: Line 119 - "many" is not needed

Response: Thank you for bringing it to our notice. We removed "many" in front of previous.

"*These four diseases were selected because they have been associated with dysphagia in previous studies."* (Line 123-124, Page 7)

Comment 14: Line 122 - the first comma before "including" is not needed

Response: Thank you for pointing out the error. We revised the text accordingly.

“Other epidemiologic factors *including* age, sex, and income, were obtained and stratified into quartiles for statistical analyses.” (Line 127-128, Page 7)

Comment 15:

1) Line 130 - perhaps instead of "from those at risk of developing dysphagia newly" could state something like "as they would not represent new/novel cases".

2) Line 131 - consider adding "dysphagia" after "incidence of" ; "The age-adjusted incidence and the incidence of [dysphagia in] each age group..."

3) Line 136 - again consider adding "dysphagia" to specify ; "The age-adjusted prevalence and prevalence [of dysphagia in] each age group was also calculated."

Response: Thank you for your valuable input. We revised the text as you suggested.

*“*People diagnosed with dysphagia before 2006 were excluded *as they would not represent new cases.* The age-adjusted incidence and the incidence of *dysphagia in* each age group were calculated for each year to control for different age distributions throughout the years. (Line 134-137, Page 8)

“The age-adjusted prevalence and prevalence *of dysphagia in* each age group were also calculated.” (Line 141-142, Page 8)

Comment 16: Line 138 and 144- I didn't catch why 2010 was written here when the title mentions data from 2006 to 2016.

Response: We are thankful for bring it to our notice. We extracted new dataset to investigate the risk of dysphagia in possible common etiologies of dysphagia and the risk of mortality in dysphagia. This was age-, sex- matched controlled data from 2010. We added a phrase to clearly describe this under statistics section.

“To investigate the risk of dysphagia in possible common etiologies of dysphagia and the risk of mortality in dysphagia, data of people who were defined as having dysphagia for the first time in 2010 (n = 39795) were extracted and these individuals were followed up until 2016; these data were compared with the data of the age- and sex-matched population without dysphagia (n = 39795).” (Line 143-147, page 8*)*

Comment 17: Line 147 - consider "a" instead of "the" when referring the Kaplan-Meier curve here.

Response: Thank you for bring it to our notice. We have revised the text under statistics section.

"Time-to-event analysis was used to estimate the mortality rate, and *a* Kaplan–Meier survival curve was plotted and compared using the log-rank test. (Line 153-155, Page 8,9)

Comment 18: Fig. 1 and 2 - Consider including the labels mentioned in lines 158-159 and 167-168 on the actual figure to simplify interpretation of the graphs

Response: Thank you for the valuable comment. We revised the figure 1 and 2 as you proposed.

Comment 19: Line 175- Consider "higher" instead of "high"

Response: Thank you for pointing it out. We revised the text accordingly.

"The risk of dysphagia was significantly *higher* in each common etiology of dysphagia, with adjustments for age, sex, low-income status, and comorbidities (Table 1)." (Line 182-183, Page 10)

Comment 20:

1) Line 181 - wording of the introductory sentence can be modified. Perhaps something like the following would make the point more easily understood: "The risk of dysphagia among the sampled comorbidities is noted from greatest to less association as follows:"

2) Line 182 - delete "s" in intervals

Response: Thank you for pointing it out. We revised the text.

"The risk of dysphagia among the sampled comorbidities is noted from greatest to less association as follows: stroke (odds ratio [OR]: 7.86, 95% confidence interval [CI]: 5.76–6.68); $p < 0.001$). (Line 188-189, Page 11)

Comment 21: Lines 187 – 189 - it makes sense how it is written but not the easiest to follow. Perhaps something like "The Kaplan-Meier plot presented in Fig. 3 demonstrates a positive correlation between dysphagia and the risk of mortality. In addition, it is shown that this mortality risk increases yearly, particularly after the first 2 years of diagnosis."

Response: Thank you for the valuable comment. We have revised the paragraph in the Results section.

"*The Kaplan-Meier plot presented in Fig. 3 demonstrates a positive correlation between dysphagia and the risk of mortality. In addition, it is shown that this mortality risk increases yearly, particularly after the first 2 years of diagnosis. (Fig. 3)."* (Line 195-197, Page 11)

Comment 22:

1) Line 200 - consider adding something like "it is shown that" after the first comma

2) Line 201-202 - perhaps consider simply stating "geriatric population" rather than numbers (60 and 70 were both used, from my review of the charts the association with the >70 age range is quite obvious but the 60s, although is perhaps statistically significant based on numbers, is not visually as obvious)

Response: Thank you for bringing it to our notice. We have revised the text accordingly in the Results section.

"In this study*, it is shown that* the overall incidence and prevalence of dysphagia increased with age. The increasing trend was conspicuous in *the geriatric population,* particularly those older than 70 years." (Line 208-210, Page 12*)*

Comment 23: Line 221 - Consider a period after 2019, and then start a next sentence starting with Cardiovascular (delete "and that").

Response: Thank you for the suggestion. However after careful consideration, we decided to retain the sentence structure since the cited reference is the same.

"*The Korean national statistics reported that medical costs for those older than 65 years were about 40% of all medical costs in 2019 and that cardiovascular disease, neoplasm, respiratory disease, and nervous system disorders were the main causes of medical expenditures."* (Line 230-233, Page 13)

Comment 24: Line 223 - Consider "demonstrates" instead of "demonstrated"

Response: Thank you for the pointing it out. We have revised the text accordingly in the Discussion section.

*"*The current study *demonstrates* that stroke, cancer, COPD, and ND significantly increase the risk of dysphagia and that the presence of dysphagia significantly increases mortality." (Line 233-235, Page 13)

Comment 25: Line 235 - consider adding "possibly" after the common following "deficits". It is known that stroke is a risk factor for dysphagia but not all strokes cause dysphagia. The essence of this point was described well in the text and does not need elaboration.

Response: Thank you for bringing it to our notice. We have revised the text accordingly in the Discussion section.

"A more severe stroke would initially have more severe neurological deficits *possibly,* including dysphagia." (Line 246-247, Page 14)

Comment 26: Line 264 and 265 - "that" are not needed

Response: Thank you for the pointing it out. We removed “that” and revised the sentence according to the citation changes under the Discussion section.

*“*Previous studies reported that the prevalence of dysphagia *ranged from 16% to 22% in* people older than 50 years*, 11.4% to 40% in geriatric population older than 65 years of age living in the community, and 25.5% to 60% in hospital/ institutional settings*.” (Line 277-280, Page 15)

Comment 27: Line 279 - "community dwellings" - I'm assuming this is a reference to the reader in regards to the nature/method of the prior studies cited earlier in the article. Perhaps here this can be reminded to the reader (this was line 204-206). I don't think this has be be a change per-se. I do believe lines 204-206 are well placed and should remain early in the discussion.

Response: Thank you for the appreciating the discussion and providing feedback.

Reviewer #3>

Comment 1:

1) The operational definition of dysphagia used requires 2 assessments in 1 month or 2 instrumental evaluation in 3 months. This could have excluded subjects with mild -moderate dysphagia who would have recovered also. Literature suggests that other than neurodegenerative disorders, others like stroke and cancer can be associated with transient rather than chronic dysphagia which also impacts short and long term outcome. Have they looked into this aspect while designing the study?

2) This exclusion of subjects with transient or short term dysphagia must have been the reason for low incidence and prevalence in their study cohort.

Response: We thank the reviewer for raising this point. In this study, we tried to define dysphagia requiring medical attention, and its prevalence, incidence, and mortality. To define persistent and unequivocal dysphagia which is related to medical practice and cost, the stringent definition and inclusion of dysphagic patients using claim code was inevitable. We thought that the single instrumental test without any dysphagia treatment or only one dysphagia therapy may mean trivial dysphagia, which may not require medical attention. According to our definition of dysphagia, even in mild or transient dysphagia, if a patient visited or is consulted for dysphagia evaluation and the medical practitioner considered him having significant dysphagia and requiring serial instrument assessment or treatment, he can be included in dysphagia group in this study. However, we agree with the reviewer that patients with mild, transient or only subjective dysphagia had high chances to be categorized to the no dysphagia group, which might be related to the lower prevalence of dysphagia seen in this study. We have added the impact of excluding mild or transient dysphagia to explain the lower prevalence of dysphagia in the discussion.

"The current study showed a relatively low prevalence of dysphagia compared to published studies….This discrepancy is probably due to our operational definition, which included patients with dysphagia who received medical attention such as outpatient or inpatient physician care, instrumental swallowing tests, and swallowing therapies….Although physician care, instrumental swallowing tests, and swallowing therapies help evaluate dysphagia accurately, they have disadvantages of cost and time compared to self-reports or standardized swallowing assessments. *In addition, people with mild or transient dysphagia may have recovered without seeing a doctor or receiving medical care, thus could not be included in the dysphagia group*." (Line 275-290, Page 15,16)

Comment 2: Have they included dysphagia assessment in only hospital settings or whether family physician assessment was also taken into account? Elderly with Presbydysphagia seems not to be included in this study cohort.

Response: We thank the reviewer for raising this point. This was a nationwide retrospective cohort study using claim code from the Korean National Health Insurance Service database. Regardless of places where the medical practices (instrumental swallowing test, swallowing therapy, NG tube insertion, percutaneous gastrostomy) were performed, the subject who met the dysphagia definition in this study could be classified to the dysphagia group. If a family

physician performed those practices and claim the costs in his clinic, those claim codes would also be recorded in NHIS database, thus dysphagia requiring medical attention could also be defined in local clinical settings. However, in line with our response to comment #1, presbyphagia which was mild or transient might not have been included in this study.

Responses to Editor's comments

Comment 1: Please remove any funding-related text from the manuscript and include it in the cover letter.

Response 1: Thank you for pointing it out. As you recommended, we have removed Funding Statement from the manuscript and included it in the cover letter.

"This work was supported by Seoul National University Bundang Hospital (https://www.snubh.org) Research Fund (No. 06-2018-140). The funders did not play a role in the study design, data collection and analysis, decision to publish, or preparation of the manuscript."

References

1. Blumenstein I, Shastri YM, Stein J. Gastroenteric tube feeding: techniques, problems and solutions. World J Gastroenterol. 2014;20(26):8505-8524.

2. Rowat A. Enteral tube feeding for dysphagic stroke patients. Br J Nurs. 2015;24(3):138, 140, 142-135.

3. Ho CH, Lin WC, Hsu YF, Lee IH, Hung YC. One-Year Risk of Pneumonia and Mortality in Patients with Poststroke Dysphagia: A Nationwide Population-Based Study. J Stroke Cerebrovasc Dis. 2018;27(5):1311-1317.

4. Good-Fratturelli MD, Curlee RF, Holle JL. Prevalence and nature of dysphagia in VA patients with COPD referred for videofluoroscopic swallow examination. Journal of communication disorders. 2000;33(2):93-110.

5. Robinson D, Jerrard-Dunne P, Greene Z, Lawson S, Lane S, O'Neill DJEGM. Oropharyngeal dysphagia in exacerbations of chronic obstructive pulmonary disease. 2011;2(4):201-3.

6. Kim JY, Kang K, Kang J, et al. Executive Summary of Stroke Statistics in Korea 2018: A Report from the Epidemiology Research Council of the Korean Stroke Society. J Stroke. 2019;21(1):42-59.
