# Supplementary material for: Trends in the incidence and prevalence of dysphagia requiring medical attention among adults in South Korea, 2006–2016: A nationwide population study
Source: PLoS One. 2023 Jun 28;18(6):e0287512. doi: 10.1371/journal.pone.0287512 (PMC10306177; doi:10.1371/journal.pone.0287512)
Supplement: Attachment — Submitted filename: Reviewer.docx [file pone.0287512.s002.docx]

**Trends in the incidence and prevalence of dysphagia requiring medical attention among adults in South Korea, 2006–2016: A nationwide population study**

**General considerations**:

- I suggest including in the first paragraph of your introduction the definition of dysphagia. It is not described anywhere. Here you can cite this paper: *Castejón-Hernández, S., Latorre-Vallbona, N., Molist-Brunet, N., Cubí-Montanyà, D., & Espaulella-Panicot, J. (2020). Association between anticholinergic burden and oropharyngeal dysphagia among hospitalized older adults. Aging Clinical and Experimental Research, 33(7), 1981–1985. doi:10.1007/s40520-020-01707-9*
- Please, do not use **elderly** to describe a population of older people. It is an ageist term. I suggest try to avoid language that might be deemed unacceptable or inappropriate (e.g. 'older people' is preferred to 'the elderly', the word 'senile' is best avoided). Take care with wording that might cause offence to ethnic or cultural groups”
- Try to avoid repetitive terms as **dysphagia** in every paragraph. Maybe you can describe your population as *dysphagic* or *non-dysphagic.* Even, you can use pronouns to refer to dysphagia in the same sentence.
- Maybe, it could be more interesting to describe **whole/general prevalence** more than a national one, because although Korea is a populous country, not all the countries have millions of population and these data could not be extrapolated into another countries.
- I am a little confused with the **main aim** of this study so maybe you should me more exact. If your objective is to know the prevalence of dysphagia independently of the cause (including mechanical, oropharyngeal, neurological … causes), **please be explicit.** In my opinion, it is not so relevant to determinate if they require medical attention or not. You only want to know a rate: the prevalence (percentage) of people affected by dysphagia
- I am not sure about if the insertion of nasogastric tube insertions should be considered as diagnostic criteria of dysphagia. What about surgical procedures? Or abdominal acute pain? Constipation?... They are clearly not related to dysphagia.
- I understand neurological disorders and cancer as a causing dysphagia… but COPD maybe is not the most important/prevalent cause of it… Please, check this bibliography which seems to be more philosophic than scientific (the journal has not impact factor).

**Specific considerations**:

Discussion:

- I propose you to add at the end of line n. 211: *“and also some side-effects from sedative drugs, opioids and also higher anticholinergic burden”.* Here you can cite Castejon et al.
- I suggest adding after “management” in line 228: “*independently of the underlying diagnosis, because in older adults the most prevalent type of dysphagia is oropharyngeal cause”.*
- In line 252, after “of dysphagia cases” it could be added the following sentence: *“even the mechanical cause of dysphagia is not the most common/prevalent in older people”.*
- If you cite the paper I recommended you before, you can include in line 266: hospital/institution setting is *“ranged from 25.5% to 55%”*.
- Consider to add at the end of line 289: *“including the review of potentially causing medications or their side effects, as sedation or xerostomia”.*
- Lines 306 and 307: Maybe they should be removed because of I explained before about the nasogastric tube.
- Lines 343 and 343: I propose to substitute “As they are common geriatric diseases and” for *“As many countries are entering an aging or super-aging society, dysphagia will be a more common problem that requires appropriate interventions in daily clinical practice such as the rest of geriatrics syndromes”.*

Thanks for your consideration! Great job but you need to continue working on your paper! Good luck!
