# Supplementary material for: Trends in the incidence and prevalence of dysphagia requiring medical attention among adults in South Korea, 2006–2016: A nationwide population study
Source: PLoS One. 2023 Jun 28;18(6):e0287512. doi: 10.1371/journal.pone.0287512 (PMC10306177; doi:10.1371/journal.pone.0287512)
Supplement: Attachment — Submitted filename: Response to Reviewers_Dysphagia_PONE 230312.docx [file pone.0287512.s004.docx]

March, 12, 2023

Sina Azadnajafabad

Academic Editors

*PLOS ONE*

Dear Editor:

We wish to re-submit the manuscript titled : “Trends in the incidence and prevalence of dysphagia requiring medical attention among adults in South Korea, 2006–2016: A nationwide population study”. The manuscript ID is PONE-D-22-35490.

We are deeply grateful to you and the reviewers of *PLOS ONE* for spending valuable time reviewing our manuscript. The comments and issues raised by the reviewers helped us improve the quality of the manuscript. We have made efforts to improve the manuscript and address the reviewers’ comments. You will find our detailed responses below and the revisions to the manuscript in red texts.

We hope that you will find the revised manuscript suitable for publication in *PLOS ONE* and look forward to receiving your response.

Sincerely,

Won-Seok Kim, MD, PhD.

Department of Rehabilitation Medicine, Seoul National University College of Medicine, Seoul National University Bundang Hospital, 166 Gumi-ro, Bundang-gu, Seongnam-si, Gyeonggi-do, 463-707, South Korea

Email: wondol77@gmail.com

Phone: +82-31-787-7737

Fax: +82-31-787-4051

**Responses to reviewer comments**

We greatly appreciate the efforts of the reviewers for their constructive comments in evaluating our manuscript. Each comment has been carefully considered and responses have been provided for each point. The revisions we made in the manuscript are written in red. The responses to the reviewers and changes in the revised manuscript are as follows.

***Reviewer #1***

**General considerations:**

**Comment 1:** I suggest including in the first paragraph of your introduction the definition of dysphagia. It is not described anywhere. Here you can cite this paper: *Castejón-Hernández, S., Latorre-Vallbona, N., Molist-Brunet, N., Cubí-Montanyà, D., & Espaulella-Panicot, J. (2020). Association between anticholinergic burden and oropharyngeal dysphagia among hospitalized older adults. Aging Clinical and Experimental Research, 33(7), 1981–1985. doi:10.1007/s40520-020-01707-9*

**Response:** We thank the reviewer for the valuable suggestion. We have added the reference and definition of dysphagia by WHO in the first paragraph of the introduction section. We also have added the citation recommended by you as Reference number 4.

*“The World Health Organization has defined dysphagia as the difficulty in forming or moving a bolus safely from the oral cavity to the esophagus and has recognized it as a medical disability associated with increased morbidity, mortality, and cost of treatment.”* (Line 50-53, Page 4)

Reference section:

*4.* *Castejón-Hernández, S., Latorre-Vallbona, N., Molist-Brunet, N., Cubí-Montanyà, D., & Espaulella-Panicot, J. (2020). Association between anticholinergic burden and oropharyngeal dysphagia among hospitalized older adults. Aging Clinical and Experimental Research, 33(7), 1981–1985. doi:10.1007/s40520-020-01707-9.*(Line 388-391, Page 21)

**Comment 2:** Please, do not use elderly to describe a population of older people. It is an ageist term. I suggest try to avoid language that might be deemed unacceptable or inappropriate (e.g. 'older people' is preferred to 'the elderly', the word 'senile' is best avoided). Take care with wording that might cause offence to ethnic or cultural groups”

**Response:** Thank you for pointing out the error in using the term “elderly”. Based on your and reviewer #2’s comments, we changed the expressions to “geriatric group” throughout the manuscript.

**Comment 3:** Try to avoid repetitive terms as dysphagia in every paragraph. Maybe you can describe your population as *dysphagic* or *non-dysphagic.* Even, you can use pronouns to refer to dysphagia in the same sentence.

**Response:** Thank you for the valuable comment. We changed the expressions throughout the manuscript.

**Comment 4:** Maybe, it could be more interesting to describe whole/general prevalence more than a national one, because although Korea is a populous country, not all the countries have millions of population and these data could not be extrapolated into another countries.

**Response:** Thank you for raising this issue. There are currently no global data on worldwide prevalence of dysphagia. Previous literature shows country-wise data on the prevalence of dysphagia among specific populations or risk groups. Therefore, we have put the greatest importance in reporting nationwide data on the incidence, prevalence, and mortality of dysphagia in Korea, and in defining the dysphagia requiring medical attention (related to medical costs or interventions) based on claim codes. We added this content to the discussion section as one of limitations.

*“Lastly, it is difficult to extrapolate the results from this study to the entire world because medical and epidemiologic circumstances of dysphagia may be different in various countries. Nevertheless, it is assumed that similar trends of incidence, prevalence, and mortality of dysphagia may be seen in aging population in developed countries where similar evaluation and treatment of dysphagia are being performed. In this regard, future studies on dysphagia using global data from various countries is necessary.”* (Line 356-362, Page 18-19)

**Comment 5:** I am a little confused with the main aim of this study so maybe you should me more exact. If your objective is to know the prevalence of dysphagia independently of the cause (including mechanical, oropharyngeal, neurological … causes), please be explicit. In my opinion, it is not so relevant to determinate if they require medical attention or not. You only want to know a rate: the prevalence (percentage) of people affected by dysphagia

**Response:** We thank the reviewer for bring up this point. In this study, we have emphasized on dysphagia requiring medical attention (related to medical interventions and costs) and analyzing its incidence, prevalence, and associations with mortality. As per the primary objective of our study, we have defined dysphagia according to the diagnostic and medical claim codes used in the Korean NHIS which is based on the ICD-10-CM. This is stated in the inclusion criteria under the material and methods section.

*“To define dysphagia, diagnostic codes and medical claim codes related to diagnosis or treatment of dysphagia were used. If a person matched at least one of the following criteria, the individual was assumed to have dysphagia: 1) presence of the diagnostic code for dysphagia (R13), 2) swallowing therapies (MX141) at least twice within 1 month, 3) instrumental swallowing tests (E7011, E7012) such as videofluoroscopic swallowing studies (VFSS) or fiberoptic endoscopic evaluation of swallowing (FEES) at least twice within 3 months, 4) nasogastric tube insertions (Q2621, Q2622) at least twice within 3 months, or 5) procedure code claims for percutaneous gastrostomy (M6730, Q2612) at least once.”* (Line 103-111, Page 6,7)

Hence any person receiving medical attention for dysphagia were categorized into dysphagia group. Therefore, we think that the term “dysphagia requiring medical attention” is the best term for specifying the objective of the study. As you have pointed out, the primary purpose is to investigate the prevalence and incidence, but our auxiliary purpose is to know the risk of dysphagia in the specific diseases which have been well-known etiologies for dysphagia and to investigate the risk of mortality due to dysphagia. We have reworded our aim and stated our primary and secondary purpose of this study at the end of the introduction section.

*“Therefore, the primary purpose of this study was to investigate the nationwide trend of the incidence and prevalence of dysphagia requiring medical attention from a large, representative, population-based cohort, using claim data from the Korean National Health Insurance Service (NHIS) database from 2006 to 2016. We further investigated the possible diseases associated with dysphagia, and the risk of long-term mortality in dysphagic individuals compared to non-dysphagic individuals.”* (Line 79-84, Page 5)

**Comment 6:** I am not sure about if the insertion of nasogastric tube insertions should be considered as diagnostic criteria of dysphagia. What about surgical procedures? Or abdominal acute pain? Constipation?... They are clearly not related to dysphagia.

**Response:** Thank you for bringing up this point. Patients who have a nasogastric tube are very likely to be unable to take sufficient food orally thus indicating obvious dysphagia. ^1,2^ As you have suggested that the one-time NG tube insertion may be due to the transient problem, so we defined the dysphagia when multiple NG tube insertions are required. Study by Ho et al. on the association of pneumonia and mortality in patients with poststroke dysphagia, also used the same definition of dysphagia for NG tube, where ≥2 nasogastric intubations was classified as dysphagia.^3^

**Comment 7:** I understand neurological disorders and cancer as a causing dysphagia… but COPD maybe is not the most important/prevalent cause of it… Please, check this bibliography which seems to be more philosophic than scientific (the journal has not impact factor).

**Response:** Thank you for the valuable comment. Stroke, neurodegenerative disease, cancer, and COPD were selected because they have been associated with dysphagia in many previous studies. Moreover, COPD is known to cause dysphagia due its dyspnea, short of breath, and related respiratory-swallowing discoordination. This is described in the discussion. As you suggested we have added 2 more studies by Good-Fratturelli, et al (Ref No. 39)^4^ and Robinson DJ,et al(Ref No. 40)^5^ to support the our viewpoint more scientifically.

Introduction section:

*“These four diseases were selected because they have been associated with dysphagia in previous studies*.” (Line 123-124, Page 7*)*

Discussion section:

*“In COPD,* *respiratory-swallowing discoordination, tachypnea and/or dyspnea, oropharyngeal swallowing dysfunction, and physical and emotional distress are associated with an increased risk of dysphagia.”* (Line 266-268, Page 14,15)

**Specific considerations**:

**Comment 8:** I propose you to add at the end of line n. 211: *“and also some side-effects from sedative drugs, opioids and also higher anticholinergic burden”.* Here you can cite Castejon et al.

**Response:** Thank you for your valuable input. We modified your suggestion a little bit for cohesiveness and added it to the text under Discussion section.

“The associated sarcopenia, frailty, pain, inadequate dentition, and polypharmacy may even aggravate the decline in swallowing function*.* *Polypharmacy in particular includes sedative drugs, opioids, and also higher anticholinergic burden, which may impair cognitive and swallowing function.”* ( Line 218-222, Page 12,13)

**Comment 9:** I suggest adding after “management” in line 228: “*independently of the underlying diagnosis, because in older adults the most prevalent type of dysphagia is oropharyngeal cause”.*

**Response:** Thank you for this suggestion. Accordingly, we added it to the text in the Discussion Section.

*“*Therefore, dysphagia in older individuals should also be considered an important cause of socioeconomic and medical burden, demanding more substantial resources and adequate screening, diagnosis, and management *independently of the underlying comorbidities because in older adults the most prevalent type of dysphagia is oropharyngeal cause.”* (Line 235-239, Page 13)

**Comment 10:** In line 252, after “of dysphagia cases” it could be added the following sentence: *“**even the mechanical cause of dysphagia is not the most common/prevalent in older people”.*

**Response:** Thank you for your input. We added it to the text in the Discussion Section.

*“*The increasing number of geriatric cancer survivors is also assumed to increase the number of dysphagia cases *even the mechanical cause of dysphagia is not the most common in older people.”* (Line 263-266, Page 14)

**Comment 11:** If you cite the paper I recommended you before, you can include in line 266: hospital/institution setting is *“ranged from 25.5% to 55%”*.

**Response:** Thank you for pointing it out. Based on your and reviewer 2’ comments, we revised the text to describe the prevalence of dysphagia in different countries in the introduction and in the discussion section. We have added the citation recommended by you as Reference number 4.

Introduction Section:

*“The prevalence of dysphagia in the geriatric population has been reported variably in different countries. In the United States, the prevalence of dysphagia in adults over 50 years of age ranges from 11.4% to 22%, and that in the geriatric population living in the community is 33%. In Europe, the prevalence of dysphagia in the geriatric population living in the community ranges from 30% to 40%, and that in hospital/ institutional settings ranges from* 25.5% to 60%. *In Asia, the prevalence of dysphagia in the geriatric population living in the community ranges from 13.8% to 33.7%.* *A meta-analysis and systematic review of the multinational studies reported that dysphagia affects 30% of community-dwelling geriatric population, almost 50% of geriatric patients, and above 50% of nursing home residents.”* (Line 61-70, Page 4,5)

Discussion Section:

*“*The current study showed a relatively low prevalence of dysphagia compared to published studies. Our study showed that the crude prevalence of dysphagia ranged from 0.09% to 0.25%. Previous studies reported that the prevalence of dysphagia *ranged from 16% to 22% in people older than 50 years, 11.4% to 40% in geriatric population older than 65 years of age living in the community, and 25.5% to 60% in hospital/ institutional settings.”* (Line 277-280, Page 15)

**Comment 12:** Consider to add at the end of line 289: *“**including the review of potentially causing medications or their side effects, as sedation or xerostomia”.*

**Response:** As per your suggestion, we have modified the sentence a little bit for cohesiveness and added it to the text under the Discussion section.

*“*As dysphagia can place a significant socioeconomic and medical burden on the Korean population, a careful analysis of the medical background is necessary to properly evaluate and treat dysphagia requiring medical attention *including the review of potentially causative diseases and medications.”* (Line 302-306, Page 16)

**Comment 13:** Lines 306 and 307: Maybe they should be removed because of I explained before about the nasogastric tube.

**Response:** After careful consideration, we decided to retain the sentence and added the description of nasogastric tube insertion in the definition criteria of dysphagia based on previous literature in the Discussion section. Also see the response to **Comment 6.**

*“In addition, the criterion of nasogastric tube insertions more than two times was used to define dysphagia in the previous study conducted in Taiwan..”* (Line 323-324, Page 17)

**Comment 14:** Lines 343 and 343: I propose to substitute “As they are common geriatric diseases and” for *“As many countries are entering an aging or super-aging society, dysphagia will be a more common problem that requires appropriate interventions in daily clinical practice such as the rest of geriatrics syndromes”.*

**Response:** Thank you for this comment. As you suggested, we revised the conclusion.

*“*The presence of stroke, ND, cancer, and COPD is associated with a high risk of dysphagia. *As many countries are entering an aging or super-aging society, dysphagia will be a more common problem that requires appropriate interventions in daily clinical practice such as the rest of geriatrics syndromes.”* (Line 366-370, Page 19)

***Reviewer #2***

**General considerations:** This article seems to represent a unique review of dysphagia based on the intended population. The article is easy to follow in its entirety. Several considerations for review/minor editing:

**Response:** Thank you for your positive words about our study. Your comments truly helped us to improve the quality of the manuscript.

**Specific considerations**

**Comment 1:** Line 22-24: perhaps these sentences can be combined: ex "The prevalence of dysphagia and the resulting socioeconomic burden are known to be increasing, here we..."

**Response:** We thank the reviewer for the suggestion, however after careful consideration, we decided to keep the two sentences separately. Combining two sentences may not reduce the number of words and that the final sentence might be too long to understand.

*The prevalence of dysphagia is increasing, resulting in socioeconomic burden, but previous reports have only been based on a specific population. Therefore, we aimed to investigate the nationwide incidence and prevalence of dysphagia requiring medical attention to provide adequate information for healthcare planning and resource allocation.* (Line 22-26, Page 2)

**Comment 2:** Line 41-42: "elderly group older than 60 years" can probably be replaced with "geriatric population" as geriatric is a term used more frequently in the paper overall

**Response:** We agree with the reviewer and have revised the expression and used “geriatric group” and “geriatric population” throughout the manuscript.

**Comment 3:**

**1)** Line 58: Perhaps "the cause of" should be "associated with" as there isn't necessarily a causal relationship demonstrated.

**2)** Line 59: "or" should be "and" ; "Indeed" is not needed.

**Response:** Thank you for pointing out the error in the text. As per your suggestion, we have revised the sentences in the introduction section.

*“*In addition, aging is *associated with* dysphagia due to muscle wasting, delayed swallowing reflex, *and* neuromuscular incoordination*. Dysphagia* is becoming more common in the *geriatric population* as life expectancy increases.” *(*Line 58-59, Page 4*)*

**Comment 4:** Lines 61-65: Reviewing the sources here, would caution using statistics from other countries given the paper here is specifically a South Korean analysis.

**Response:** We thank the reviewer for raising this point. This study used data of South Korean population specifically. So, we revised the text as you suggested to clearly describe where the citations come from. Furthermore, we tried to clearly describe where the country-wise statistics come from (e.g., Europe, US,etc.) in the introduction section..

*“Dysphagia is becoming more common in the geriatric population as life expectancy increases. The prevalence of dysphagia in the geriatric population has been reported variably in different countries. In the United States, the prevalence of dysphagia in adults over 50 years of age ranges from 11.4% to 22%, and that in the geriatric population living in the community is 33%. In Europe, the prevalence of dysphagia in the geriatric population living in the community ranges from 30 to 40%, and that in hospital/ institutional settings ranges from 25.5% to 60%. In Asia, the prevalence of dysphagia in the geriatric population living in the community ranges from 13.8% to 33.7%. A meta-analysis and systematic review of the multinational studies reported that dysphagia affects 30% of community-dwelling geriatric population, almost 50% of geriatric patients, and above 50% of nursing home residents.”* (Line 60-70, Page 4,5)

**Comment 5:** Line 66: “However” is not needed

**Response:** Thank you for bringing it to our notice. We have removed “However,” and revised the text in the introduction section.

*“*The previously reported prevalence of dysphagia is not representative of the entire population because most of these reports have come from cross-sectional studies with small sample sizes, specific diseases (e.g., stroke, ND, cancer), and specific healthcare settings (hospitalized/institution settings) with specific age groups (healthy or frail *geriatric population).”* (Line 72-76, Page 5)

**Comment 6:**

**1)** Line 73: Consider adding "the" after of.

**2)** Line 74: Perhaps instead of "in a large, representative," can simply state "from a". It is assumed a population database has those characteristics.

**Response:** As per your suggestion, we have revised the text in the Introduction section.

“Therefore, *the primary purpose of this study was* to investigate the nationwide trend of *the* incidence and prevalence of dysphagia requiring medical attention *from* a large, representative, population-based cohort, using claim data from the Korean National Health Insurance Service (NHIS) database from 2006 to 2016.” (Line 79-82, Page 5)

**Comment 7:**

**1)** Line 78: "Therefore" not needed.

**2)** Line 80: "Thus" not needed

**Response:** As per your suggestion, we deleted “Therefore” at the beginning of the sentence and “thus” in front of emphasizing. However, after careful consideration, we decided to remove this final sentence of the introduction, in order to present the study purpose concisely.

#Line 84-88, Page 5 (removed): *This study aimed to present clear nationwide evidence on the prevalence, incidence, and mortality of dysphagia in South Korea, emphasizing the health-related impact of dysphagia in an aging society to provide adequate information for healthcare planning and resource allocation.*

**Comment 8:**

**1)** Line 87: consider "universal" instead of "mandatory"; at least in the United States the term "universal healthcare" is used, I can't comment on Canada or Europe.

**2)** Line 89: consider adding an ambiguous word such as "presumptively" after NHIS; this article makes a point of highlighting the weakness of the reporting system in the discussion. It is doubtfully a perfect system.

**Response:** Thank you for bringing it to our notice. We have revised the text in the materials and methods section.

*“*The NHIS is a *universal* social health insurance that provides healthcare coverage and medical aid to most of the Korean population (97.2% and 2.8%, respectively). Therefore, the NHIS *presumptively* has all records of medical service claims and sociodemographic information of the entire Korean population*.”* (Line 89-93, Page 6)

**Comment 9:** Line 100 (the entire section): The parameters investigated were inclusive and well described.

**Response:** We would like to thank the reviewer for appreciating the criteria.

**Comment 10:** Lines 112-117: Would use the same formatting used earlier in the section: replace the period after dysphagia in line 113 with a colon (:), then use commas (,) instead of semicolons (;) before "2)" and "3)" and "and 4)".

**Response:** Thank you for bringing it to our notice. We have revised the paragraph according to your **comment 10** and **comment 11**.

*“*Medical claim codes based on ICD-10-CM were used to define the diseases that may have caused dysphagia: 1) stroke as diagnostic codes of I63 and I64 and brain imaging (computed tomography or magnetic resonance imaging) during admission, 2) ND as diagnostic codes claimed for Parkinson’s disease (G20 and V124) or those claimed for dementia (F00-03, G30, G31, G231, G310, G318, and F107), 3) cancer as diagnostic code of C codes *(C00-14, C15-25, C32-34, C50, C53, C54, C56, C61, C62, C64, C67, C70-72, C73, C81-86, C90-95*) and V193, and 4) chronic obstructive pulmonary disease (COPD) as diagnostic codes of J41-J44. V193 and V194 are specific deductible insurance codes for cancer and Parkinson's disease respectively *in Korea.* These four diseases were selected because they have been associated with dysphagia in previous studies.” (Line 114-124, Page 7)

**Comment 11:** Lines 113-114 - The context of the sentence sounds like something was edited out or was intended to be removed but was kept. Were there also codes used for specific brain imaging to identify stroke that were supposed to be given ICD-10 codes?

**Response:** Thank you for the raising this point. In line 115-116, we intended to specifically define ischemic or hemorrhagic stroke patients, whose diagnosis was confirmed with not only ICD-10-CM codes but also with brain lesions on imaging studies such as brain CT or MRI. In the previous study, defining stroke using ICD-10 codes alone was not valid, and defining stroke using ICD-10 codes and medical claims of brain CT or MRI was more valid for estimating the prevalence of stroke (Kim et al.).^6^

**Comment 12:** Line 116 - perhaps in this section "cancer" should be described more. It was in fact elaborated on in the discussion, but it might be more consistent to given an explanation here, as this is the definitions section of the article.

**Response:** We thank the reviewer to the suggestion. The C codes (C00.00-C96) from ICD-10-CM includes various types of neoplasm, and we chose specific codes among them: oral cavity and pharynx (C00–C14), digestive organs (C15-25), respiratory and intrathoracic organs (C32-34), skin (C43), breast (C50), genital organs (C53, C54, C56, C61, C62), urinary tract (C64, C67), central nervous system (C70–72), thyroid (C73), lymphoma (C81–86), multiple myeloma (C90), and leukemia (C91–95). And V193 and V194 are specific deductible insurance codes for cancer and Parkinson's disease respectively in Korea.

We added this explanation in the operational definition under the Material and methods section.

*“* Medical claim codes based on ICD-10-CM were used to define the diseases that may have caused dysphagia: 1) stroke as diagnostic codes of I63 and I64 and brain imaging (computed tomography or magnetic resonance imaging) during admission, 2) ND as diagnostic codes claimed for Parkinson’s disease (G20 and V124) or those claimed for dementia (F00-03, G30, G31, G231, G310, G318, and F107), 3) cancer as diagnostic code of C codes *(C00-14, C15-25, C32-34, C50, C53, C54, C56, C61, C62, C64, C67, C70-72, C73, C81-86, C90-95)* and V193, and 4) chronic obstructive pulmonary disease (COPD) as diagnostic codes of J41-J44*. V193 and V194 are specific deductible insurance codes for cancer and Parkinson's disease respectively in Korea.* These four diseases were selected because they have been associated with dysphagia in previous studies.” (Line 114-124, Page 7)

**Comment 13:** Line 119 - "many" is not needed

**Response:** Thank you for bringing it to our notice. We removed “many” in front of previous.

“*These four diseases were selected because they have been associated with dysphagia in previous studies.”* (Line 123-124, Page 7)

**Comment 14:** Line 122 - the first comma before "including" is not needed

**Response:** Thank you for pointing out the error. We revised the text accordingly.

“Other epidemiologic factors *including* age, sex, and income, were obtained and stratified into quartiles for statistical analyse*s*.” (Line 127-128, Page 7)

**Comment 15:**

**1)** Line 130 - perhaps instead of "from those at risk of developing dysphagia newly" could state something like "as they would not represent new/novel cases".

**2)** Line 131 - consider adding "dysphagia" after "incidence of" ; "The age-adjusted incidence and the incidence of [dysphagia in] each age group..."

**3)** Line 136 - again consider adding "dysphagia" to specify ; "The age-adjusted prevalence and prevalence [of dysphagia in] each age group was also calculated."

**Response:** Thank you for your valuable input. We revised the text as you suggested.

*“*People diagnosed with dysphagia before 2006 were excluded *as they would not represent new cases.* The age-adjusted incidence and the incidence of *dysphagia in* each age group were calculated for each year to control for different age distributions throughout the years. (Line 134-137, Page 8)

“The age-adjusted prevalence and prevalence *of dysphagia in* each age group were also calculated.” (Line 141-142, Page 8)

**Comment 16:** Line 138 and 144- I didn't catch why 2010 was written here when the title mentions data from 2006 to 2016.

**Response:** We are thankful for bring it to our notice. We extracted new dataset to investigate the risk of dysphagia in possible common etiologies of dysphagia and the risk of mortality in dysphagia. This was age-, sex- matched controlled data from 2010. We added a phrase to clearly describe this under statistics section.

*“To investigate the risk of dysphagia in possible common etiologies of dysphagia and the risk of mortality in dysphagia,* data of people who were defined as having dysphagia for the first time in 2010 (n = 39795) were extracted and these individuals were followed up until 2016; these data were compared with the data of the age- and sex-matched population without dysphagia (n = 39795).” (Line 143-147, page 8*)*

**Comment 17:** Line 147 - consider "a" instead of "the" when referring the Kaplan-Meier curve here.

**Response:** Thank you for bring it to our notice. We have revised the text under statistics section.

“Time-to-event analysis was used to estimate the mortality rate, and *a* Kaplan–Meier survival curve was plotted and compared using the log-rank test*.* (Line 153-155, Page 8,9)

**Comment 18:** Fig. 1 and 2 - Consider including the labels mentioned in lines 158-159 and 167-168 on the actual figure to simplify interpretation of the graphs

**Response:** Thank you for the valuable comment. We revised the figure 1 and 2 as you proposed.

**Comment 19:** Line 175- Consider "higher" instead of "high"

**Response:** Thank you for pointing it out. We revised the text accordingly.

“The risk of dysphagia was significantly *higher* in each common etiology of dysphagia, with adjustments for age, sex, low-income status, and comorbidities (Table 1).” (Line 182-183, Page 10)

**Comment 20:**

**1)** Line 181 - wording of the introductory sentence can be modified. Perhaps something like the following would make the point more easily understood: "The risk of dysphagia among the sampled comorbidities is noted from greatest to less association as follows:"

**2)** Line 182 - delete "s" in intervals

**Response:** Thank you for pointing it out. We revised the text.

*“The risk of dysphagia among the sampled comorbidities is noted from greatest to less association as follows: stroke (odds ratio [OR]: 7.86,* *95% confidence interval [CI]: 5.76–6.68); p < 0.001*). (Line 188-189, Page 11)

**Comment 21:** Lines 187 – 189 - it makes sense how it is written but not the easiest to follow. Perhaps something like "The Kaplan-Meier plot presented in Fig. 3 demonstrates a positive correlation between dysphagia and the risk of mortality. In addition, it is shown that this mortality risk increases yearly, particularly after the first 2 years of diagnosis."

**Response:** Thank you for the valuable comment. We have revised the paragraph in the Results section.

“*The Kaplan-Meier plot presented in Fig. 3 demonstrates a positive correlation between dysphagia and the risk of mortality. In addition, it is shown that this mortality risk increases yearly, particularly after the first 2 years of diagnosis. (Fig. 3).”* (Line 195-197, Page 11)

**Comment 22:**

**1)** Line 200 - consider adding something like "it is shown that" after the first comma

**2)** Line 201-202 - perhaps consider simply stating "geriatric population" rather than numbers (60 and 70 were both used, from my review of the charts the association with the >70 age range is quite obvious but the 60s, although is perhaps statistically significant based on numbers, is not visually as obvious)

**Response:** Thank you for bringing it to our notice. We have revised the text accordingly in the Results section.

“In this study*, it is shown that* the overall incidence and prevalence of dysphagia increased with age. The increasing trend was conspicuous in *the geriatric population,* particularly those older than 70 years.” (Line 208-210, Page 12*)*

**Comment 23:** Line 221 - Consider a period after 2019, and then start a next sentence starting with Cardiovascular (delete "and that").

**Response:** Thank you for the suggestion. However after careful consideration, we decided to retain the sentence structure since the cited reference is the same.

“*The Korean national statistics reported that medical costs for those older than 65 years were about 40% of all medical costs in 2019 and that cardiovascular disease, neoplasm, respiratory disease, and nervous system disorders were the main causes of medical expenditures.”* (Line 230-233, Page 13)

**Comment 24:** Line 223 - Consider "demonstrates" instead of "demonstrated"

**Response:** Thank you for the pointing it out. We have revised the text accordingly in the Discussion section.

*“*The current study *demonstrates* that stroke, cancer, COPD, and ND significantly increase the risk of dysphagia and that the presence of dysphagia significantly increases mortality.” (Line 233-235, Page 13)

**Comment 25:** Line 235 - consider adding "possibly" after the common following "deficits". It is known that stroke is a risk factor for dysphagia but not all strokes cause dysphagia. The essence of this point was described well in the text and does not need elaboration.

**Response:** Thank you for bringing it to our notice. We have revised the text accordingly in the Discussion section.

“A more severe stroke would initially have more severe neurological deficits *possibly,* including dysphagia.” (Line 246-247, Page 14)

**Comment 26:** Line 264 and 265 - "that" are not needed

**Response:** Thank you for the pointing it out. We removed “that” and revised the sentence according to the citation changes under the Discussion section.

*“*Previous studies reported that the prevalence of dysphagia *ranged from 16% to 22% in* people older than 50 years*, 11.4% to 40% in geriatric population older than 65 years of age living in the community, and 25.5% to 60% in hospital/ institutional settings*.” (Line 277-280, Page 15)

**Comment 27:** Line 279 - "community dwellings" - I'm assuming this is a reference to the reader in regards to the nature/method of the prior studies cited earlier in the article. Perhaps here this can be reminded to the reader (this was line 204-206). I don't think this has be be a change per-se. I do believe lines 204-206 are well placed and should remain early in the discussion.

**Response:** Thank you for the appreciating the discussion and providing feedback.

***Reviewer #3>***

**Comment 1:**

**1)** The operational definition of dysphagia used requires 2 assessments in 1 month or 2 instrumental evaluation in 3 months. This could have excluded subjects with mild -moderate dysphagia who would have recovered also. Literature suggests that other than neurodegenerative disorders, others like stroke and cancer can be associated with transient rather than chronic dysphagia which also impacts short and long term outcome. Have they looked into this aspect while designing the study?

**2)** This exclusion of subjects with transient or short term dysphagia must have been the reason for low incidence and prevalence in their study cohort.

**Response:** We thank the reviewer for raising this point. In this study, we tried to define dysphagia requiring medical attention, and its prevalence, incidence, and mortality. To define persistent and unequivocal dysphagia which is related to medical practice and cost, the stringent definition and inclusion of dysphagic patients using claim code was inevitable. We thought that the single instrumental test without any dysphagia treatment or only one dysphagia therapy may mean trivial dysphagia, which may not require medical attention. According to our definition of dysphagia, even in mild or transient dysphagia, if a patient visited or is consulted for dysphagia evaluation and the medical practitioner considered him having significant dysphagia and requiring serial instrument assessment or treatment, he can be included in dysphagia group in this study. However, we agree with the reviewer that patients with mild, transient or only subjective dysphagia had high chances to be categorized to the no dysphagia group, which might be related to the lower prevalence of dysphagia seen in this study. We have added the impact of excluding mild or transient dysphagia to explain the lower prevalence of dysphagia in the discussion.

*“*The current study showed a relatively low prevalence of dysphagia compared to published studies….This discrepancy is probably due to our operational definition, which included patients with dysphagia who received medical attention such as outpatient or inpatient physician care, instrumental swallowing tests, and swallowing therapies….Although physician care, instrumental swallowing tests, and swallowing therapies help evaluate dysphagia accurately, they have disadvantages of cost and time compared to self-reports or standardized swallowing assessments. *In addition, people with mild or transient dysphagia may have recovered without seeing a doctor or receiving medical care, thus could not be included in the dysphagia group*.” (Line 275-290, Page 15,16)

**Comment 2:** Have they included dysphagia assessment in only hospital settings or whether family physician assessment was also taken into account? Elderly with Presbydysphagia seems not to be included in this study cohort.

**Response:** We thank the reviewer for raising this point. This was a nationwide retrospective cohort study using claim code from the Korean National Health Insurance Service database. Regardless of places where the medical practices (instrumental swallowing test, swallowing therapy, NG tube insertion, percutaneous gastrostomy) were performed, the subject who met the dysphagia definition in this study could be classified to the dysphagia group. If a family physician performed those practices and claim the costs in his clinic, those claim codes would also be recorded in NHIS database, thus dysphagia requiring medical attention could also be defined in local clinical settings. However, in line with our response to comment #1, presbyphagia which was mild or transient might not have been included in this study.

**Responses to Editor’s comments**

**Comment 1**: Please remove any funding-related text from the manuscript and include it in the cover letter.

**Response 1:** Thank you for pointing it out. As you recommended, we have removed Funding Statement from the manuscript and included it in the cover letter.

*"This work was supported by Seoul National University Bundang Hospital (*[*https://www.snubh.org*](https://www.snubh.org/)*) Research Fund (No. 06-2018-140). The funders did not play a role in the study design, data collection and analysis, decision to publish, or preparation of the manuscript."*

**References**

1.	Blumenstein I, Shastri YM, Stein J. Gastroenteric tube feeding: techniques, problems and solutions. World J Gastroenterol*.* 2014;20(26):8505-8524.

2.	Rowat A. Enteral tube feeding for dysphagic stroke patients. Br J Nurs. 2015;24(3):138, 140, 142-135.

3.	Ho CH, Lin WC, Hsu YF, Lee IH, Hung YC. One-Year Risk of Pneumonia and Mortality in Patients with Poststroke Dysphagia: A Nationwide Population-Based Study. J Stroke Cerebrovasc Dis. 2018;27(5):1311-1317.

4.	Good-Fratturelli MD, Curlee RF, Holle JL. Prevalence and nature of dysphagia in VA patients with COPD referred for videofluoroscopic swallow examination. Journal of communication disorders. 2000;33(2):93-110.

5.	Robinson D, Jerrard-Dunne P, Greene Z, Lawson S, Lane S, O’Neill DJEGM. Oropharyngeal dysphagia in exacerbations of chronic obstructive pulmonary disease. 2011;2(4):201-3.

6.	Kim JY, Kang K, Kang J, et al. Executive Summary of Stroke Statistics in Korea 2018: A Report from the Epidemiology Research Council of the Korean Stroke Society. J Stroke. 2019;21(1):42-59.
