# Supplementary material for: Trends in the incidence and prevalence of dysphagia requiring medical attention among adults in South Korea, 2006–2016: A nationwide population study
Source: PLoS One. 2023 Jun 28;18(6):e0287512. doi: 10.1371/journal.pone.0287512 (PMC10306177; doi:10.1371/journal.pone.0287512)
Supplement: Attachment — Submitted filename: Response to Reviewers_Dysphagia_PONE 230524.docx [file pone.0287512.s006.docx]

May, 24, 2023

Sina Azadnajafabad

Academic Editors

*PLOS ONE*

Dear Editor:

We wish to re-submit the manuscript titled : “Trends in the incidence and prevalence of dysphagia requiring medical attention among adults in South Korea, 2006–2016: A nationwide population study”. The manuscript ID is PONE-D-22-35490.

We are deeply grateful to you and the reviewers of *PLOS ONE* for spending valuable time reviewing our manuscript. The comments and issues raised by the reviewers helped us improve the quality of the manuscript. We have made efforts to improve the manuscript and address the reviewers’ comments. You will find our detailed responses below and the revisions to the manuscript in red texts.

We hope that you will find the revised manuscript suitable for publication in *PLOS ONE* and look forward to receiving your response.

Sincerely,

Won-Seok Kim, MD, PhD.

Department of Rehabilitation Medicine, Seoul National University College of Medicine, Seoul National University Bundang Hospital, 166 Gumi-ro, Bundang-gu, Seongnam-si, Gyeonggi-do, 463-707, South Korea

Email: wondol77@gmail.com

Phone: +82-31-787-7737

Fax: +82-31-787-4051

**Responses to reviewer comments**

We greatly appreciate the efforts of the reviewers for their constructive comments in evaluating our manuscript. Each comment has been carefully considered and responses have been provided for each point. The line numbers of the comments and responses are refer to the marked up version of the re-submitted manuscript. The revisions we made in the manuscript are written in red. The responses to the reviewers and changes in the revised manuscript are as follows.

***Reviewer #1***

**Comment 1:** Change the expression “geriatric group” to “geriatric population” in the abstract.

**Response:** Thank you for pointing out. We have changed the expression in the abstract.

*“The increasing trend was conspicuous in the geriatric population.”* (Line 40, Page 2)

**Comment 2:** Change the expression “and” to “or” in Line 55.

**Response:** Thank you for pointing it out. We have changed the term in the manuscript.

*“The swallowing process can be disrupted by diverse diseases such as stroke, traumatic brain injury, neurodegenerative disease (ND), motor neuron diseases, or cancers.”* (Line 54-56, Page 4)

***Reviewer #2***

**Comment 1:** Line 143 to 150 - It still does not make sense why there is a reference to the year 2010. The title would seem to indicate data would have been taken from 2006 to 2016. Why wasn't 2006 written in lines 145 and 150 instead of 2010? On line 96 it states that data was taken from 2006 to 2016. Perhaps a simple comment to clarify this for the reader should be added. I don't think most readers would notice this issue. Refer to comment 16 to reviewer 2 from the original submission.

**Response**: Thank you for your comments. We have conducted an analysis of the incidence and prevalence of dysphagia from 2006 to 2016. However, in order to investigate the risk of dysphagia in potential common etiologies, we specifically constructed a cross-sectional dataset in 2010. The reason for selecting this particular year is twofold: firstly, we believe that the dysphagia practices utilizing the claim codes used for dysphagia definition would have been stable during this period, ensuring reliable data; secondly, the data from 2010 provides a suitable cohort for long-term mortality follow-up. We have included the following paragraphs in the manuscript and believe that the readers will comprehend the utilization and purpose of the cross-sectional dataset in 2010.

*“To investigate the risk of dysphagia in possible common etiologies of dysphagia and the risk of mortality in dysphagia, data of people who were defined as having dysphagia for the first time in 2010 (n = 39795) were extracted and these individuals were followed up until 2016; these data were compared with the data of the age- and sex-matched population without dysphagia (n = 39795).*” (Line 143-147, page 8*)*

**Comment 2:** Line 22 - consider "limited" instead of "specific" and add "s" after population ("populationS")

**Response:** “*The prevalence of dysphagia is increasing, resulting in socioeconomic burden, but previous reports have only been based on a limited populations.”* (Line 22, Page 2)

**Comment 3:** Line 40 to 42 - consider deleting the sentence starting with "The presence of stroke...". This is repetitive to what is already mentioned in lines 34-37

**Response:** Thank you for the suggestion. However after careful consideration, we have decided to retain the sentence. We think Line 39-42 is necessary to conclude the abstract with highlighting the main findings of the current study and emphasizing the importance of dysphagia in the geriatric healthcare. (Line 41-42, Page 2)

**Comment 4:** Line 46-47 - consider deleting this sentence it is repetitive/doesn't add anything new

**Response:** Thank you for the suggestion. However after careful consideration, we have decided to retain the sentence. In the current study, the operational definition of swallowing included both oropharyngeal and esophageal dysphagia, and we tried to clarify in the beginning that the swallowing process involves various phases from the oral cavity to the stomach.

*“Swallowing is the process of passing solid and liquid food from the oral cavity, pharynx, and esophagus into the stomach with appropriate coordination.”* (Line 46-47, page 4)

**Comment 5:** Line 51 - consider adding "," after [4]

**Response:** *“The World Health Organization has defined dysphagia... esophagus [4], and has recognized it as a medical disability associated with increased morbidity, mortality, and cost of treatment [1,3]”* (Line 50-53, Page 4)

**Comment 6:** Line 56 - consider changing "with the aging of the population" to "with an aging population" - this would sound more in line with the rest of the article

**Response: “***Since these diseases are increasing with an aging of the population, the incidence of dysphagia....*” (Line56, Page 4)

**Comment 7:** Line 215 - consider adding "and comorbidities" after "geriatric syndromes".

**Response:** *“The relationship between dysphagia and aging can first be explained by geriatric syndromes and comorbidities.”* (Line 214-215, Page 12)

**Comment 8:** Line 215 - 218 - the sentence following "[7-8]" doesn't really contribute the the text, but doesn't hurt it either. I would consider deleting it to shorten the discussion.

**Response:** Thank you for the suggestion. However after careful consideration, we decided to retain the sentence because the description of the changes in swallowing physiology in the geriatric population could explain the relationship between aging and dysphagia; besides, this also explains the conspicuous increase of incidence and prevalence of dysphagia in the geriatric population in the result section.

*“The swallowing function may decline with aging, followed by changes in swallowing physiology, such as weakness of the swallowing reflex, cough response, bolus propulsion, and increased residue.”* (Line 216-218, Page 12)

**Comment 9:** Line 238 - Consider adding "," after "because"

**Response:***“Therefore, dysphagia in older individuals... independently of the underlying comorbidities, because in older adults the most prevalent type of dysphagia is oropharyngeal cause.”* (Line 235-239, Page 13)

**Comment 10:** Line241 - Consider changing "were" to "are"

**Response:** Thank you for the suggestion. However after careful consideration, we decided to retain “were” to match the tense of the preceding phrase describing the analysis of the current study.

*“Additionally, dysphagia burden may be highlighted because this analysis was conducted using the NHIS claim codes, including objectively defined dysphagia and etiologies, which were related to medical expenditure.”* (Line 239-242, Page 13)

**Comment 11:** Line 247 - Consider moving "possibly" after the ","

**Response:** *“A more severe stroke would initially have more severe neurological deficits, possibly including dysphagia.”* (Line 246-247, Page 14)

**Comment 12:** Line 256- Consider changing "depend" to "depends"

**Response:** Thank you for the suggestion. However after careful consideration, we decided to retain “depend” because the subject of the sentence (“the mechanisms of dysphagia”) is plural.

*“In dementia, the mechanisms of dysphagia depend on the type and progression of dementia.”* (Line 255-257, Page 14)

**Comment 13:** Line 261- Consider splitting the sentence into two, the most logical way would be to add a "." after "food passage", delete "and" and start the next sentence with "Treatment modalities..."

**Response:** *“Cancers, particularly of the head and neck and upper gastrointestinal tract, may interfere with food passage. Treatment modalities... swallowing function.”* (Line 260-263, Page 14)

**Comment 14:** Line 265- Consider ending the sentence with "." after "dysphagia cases" and deleting the rest of the sentence. I'm not sure what was trying to be said, but it likely was not necessary or adding anything important to the article.

**Response:** *“The increasing number of geriatric cancer survivors is also assumed to increase the number of dysphagia cases.”* ~~even the mechanical cause of dysphagia is not the most common in older people~~ (Line 263-265, Page 14)

**Comment 15:** Line 276- Consider removing "our study showed that" to reduce repetition

**Response:** *“The current study showed a relatively low prevalence of dysphagia compared to published studies~~. Our study showed that~~ The crude prevalence of dysphagia ranged from 0.09% to 0.25%.”* (Line 275-277, Page 15)

**Comment 16:** Line 305- Consider adding "," after "medical attention"

**Response:** *“As dysphagia can place… dysphagia requiring medical attention, including the review of potentially causative diseases and medications.”* (Line 302-306, page 16)

**Comment 17:** Line 317- Consider changing "the instrumental" to "an instrumental" and making tests singular (tests -> test).

**Response:** *“When a penetration or aspiration is detected by an instrumental swallowing test~~s~~....”* (Line 316-317, page 17)

**Comment 18:**

**1)** Line 324- Consider changing "dysphagia in the previous" to "dysphagia in a previous"

**2)** Line 324- Consider adding something like ", and we utilized this as well." after Taiwan.

**Response:** *“In addition, the criterion of nasogastric tube insertions more than two times was used to define dysphagia, as similarly described in a previous study conducted in Taiwan.”* (Line 323-325 Page 17)

**Comment 19:** Line 359- Consider changing "seen in aging population in developed" to "seen in the aging populations of other"

**Response:** *“Nevertheless, it is assumed that similar trends of incidence, prevalence, and mortality of dysphagia may be seen in the aging population in other countries where similar evaluation and treatment of dysphagia are being performed.”* (Line 358-360, Page 18-19)

**Comment 20:** Line 360- Consider ending the sentence after "countries" and deleting the rest of the sentence.

**Response:** Thank you for the suggestion. However after careful consideration, we decided to retain the phrase. The last paragraph of the discussion describes that the results from this study are hard to extrapolate to the entire world. Therefore, it will be more persuasive for the reader to state in the last paragraph the results of the current study may be seen in other countries with similar medical and epidemiologic circumstances.

*“Nevertheless, it is assumed that similar trends of incidence, prevalence, and mortality of dysphagia may be seen in the aging population in other countries where similar evaluation and treatment of dysphagia are being performed.”* (Line 358-360, Page 18-19)

***To Editor office:***

Please note that we have changed the financial disclosure; funding award information from “No. 06-2018-140” to “No. 06-2018-0184.”
